# Nanoscale Observation of Nickel(II) Sequestration by Green Rust Sulfate

**DOI:** 10.1021/acsearthspacechem.5c00199

**Published:** 2025-10-28

**Authors:** Khondaker M.N. Alam, Junho Han, Bojeong Kim, Evert J. Elzinga

**Affiliations:** 1 Department of Earth & Environmental Sciences, 67206 Rutgers University, 101 Warren Street, Newark, New Jersey 07102, United States; 2 Environmental Planning Institute, 26725 Seoul National University, Gwanak-ro 1, Seoul 08826, The Republic of Korea; 3 Department of Earth and Environmental Science, Temple University, Philadelphia, Pennsylvania 19122, United States

**Keywords:** nickel, green rust, iron, soil, coprecipitation, aging, sorption, solubility

## Abstract

Green rust (GR) is a mixed-valent Fe-layered double hydroxide (Fe­(II)–Fe­(III)-LDH) mineral that is prevalent in reducing geochemical environments, where it exhibits high sorption and redox reactivity. Here, we examined the morphology and chemistry of individual GR particles with nanoscale resolution in the presence and absence of Ni­(II)_aq_ at reaction times between 1 h and 3 months using scanning transmission electron microscopy coupled with energy dispersive X-ray spectroscopy (STEM-EDXS). During the first day of reaction, sorbed Ni­(II) accumulated alongside Fe in ∼10 nm thick rims around the GR particle edges, consistent with the formation of mixed Ni­(II)/Fe­(II)–Fe­(III)-LDH. After 3 months, the rims were thinner and contained less Ni­(II) despite a doubling of the sorbed load, suggesting sequestration of Ni­(II) sorbates into the bulk during aging. Sequential extractions similarly provided evidence for declining levels of sorbed Ni­(II) at the GR surface with time, concurrent with increasing levels inside the mineral bulk. The combined STEM-EDXS and extraction results demonstrate chemical and structural variations across GR particle surfaces and redistribution of Ni­(II) sorbates during aging over time scales of days–weeks. These findings provide new mechanistic insights into the processes controlling the partitioning and mobility of trace metals in reducing geochemical systems.

## Graphical abstract

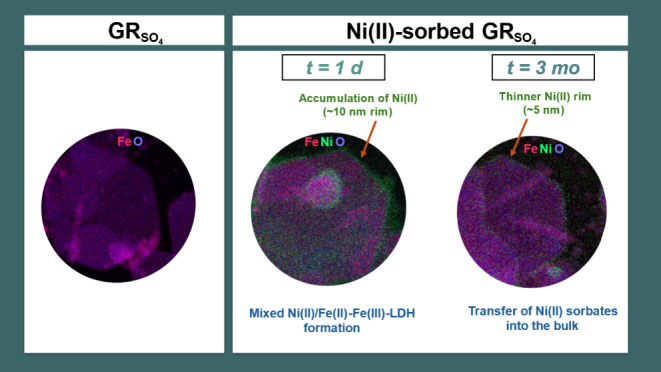

## Introduction

Green rust (GR) is a mixed-valent Fe­(II)/Fe­(III) layered double hydroxide (Fe­(II)–Fe­(III)-LDH) mineral that is common in reducing geochemical systems such as flooded soils, suboxic groundwaters, corroding drinking water lines, permeable reactive barriers, and iron electrocoagulation water treatments.^1–14^ The mineral is composed of positively charged octahedral sheets that are charge-balanced by anions hosted in the hydrated interlayers.^1,14^ GR possesses a large specific surface area and may interact with a broad range of trace elements through redox^1,15–24^ and sorption reactions.^1,25–31^ As a result, it exerts considerable influence on the biogeochemical cycling and mobility of pollutants and nutrients in reducing environments.^1,30,32–34^ While other Fe-bearing phases, including ferrihydrite, goethite, Fe­(II)-silicates, and sulfide minerals, are also abundant in suboxic environments and may strongly influence the mobility of contaminants and nutrients through sorption and redox processes.^35–37^ GR is of particular interest because of its unique structure, properties, and reactivity in reducing environments. However, the mechanisms governing trace metal sorption onto this mineral remain underexplored, and this is especially relevant for Ni­(II), which is widely released from mining, electroplating, and steel manufacturing and frequently detected in groundwater and soils.^38,39^ Such settings often overlap with conditions favorable for GR formation, underscoring the importance of understanding Ni-GR interactions in reducing environments.^40,41^

In our previous studies,^27,28^ we investigated the sorption of a series of common redox-stable divalent metal [Me­(II)] species [Mn­(II), Co­(II), Ni­(II), Zn­(II), and Cd­(II)] with GR using a combination of batch kinetic experiments and X-ray absorption spectroscopic (XAS) analyses, and observed considerable differences in the extent and mode of Me­(II) uptake. The sorption of Mn­(II) and Cd­(II) was limited and confined to Me­(II)-Fe­(II) cation exchange at the GR surface. Much more extensive sorption occurred for Co­(II), Ni­(II), and Zn­(II), which coprecipitated with Fe­(II) and Fe­(III) to form mixed Me­(II)/Fe­(II)–Fe­(III)-LDH phases. The combined batch and spectroscopic data suggested that the mechanism of coprecipitation was time-dependent, with coupled Me­(II)-Fe­(II) cation exchange and coprecipitation reactions dominating the early stages of sorption, and sorption at later stages driven by Fe­(II)-catalyzed recrystallization or solubility differences between GR and the secondary Me­(II)-LDH phases.^27,28^ This mechanistic and temporal complexity is important to the fate of trace metals in reducing systems such as suboxic soils, where reductive dissolution of Fe­(III)-oxides establishes geochemical conditions favorable to the formation of GR and leads to the release of trace metal sorbates to solution.^42–45^

The bulk EXAFS analyses of our earlier studies helped constrain the sorption mechanisms of Co­(II), Ni­(II), and Zn­(II) with GR, but a conclusive determination was not possible. A more detailed characterization is of interest, as the mechanisms proposed will yield distinct Me­(II) sorption products. Cation exchange and coprecipitation reactions will accumulate Me­(II) sorbates in Me­(II)/Fe­(II)–Fe­(III)-LDH phases at or near the GR surface, whereas Me­(II) substitution during Fe­(II)-catalyzed GR recrystallization will lead to the formation of dilute Me­(II):GR solid solutions with Me­(II) sorbates dispersed diffusely throughout the sorbent lattice.^27,28,46,47^ These differences in coordination and spatial distribution cannot be resolved with bulk EXAFS, but likely control the stability of sorbed Me­(II) and its susceptibility to desorption. Further studies complementing the bulk EXAFS work conducted thus far are therefore needed.

Here, we employed scanning transmission electron microscopy coupled with energy-dispersive X-ray spectroscopy (STEM-EDXS) to characterize the spatial distribution of sorbed Ni­(II) at the GR surface. The nanoscale spatial resolution of STEM-EDXS is well suited for elemental mapping of GR mineral particles, which typically are of sub-μm size.^48–50^ Moreover, the 2-dimensional (i.e., platelet) structure of GR crystals permits distinction between the positively charged basal planes, which are inert to metal cation sorption, and the crystal edges, which contain amphoteric surface sites that are involved in metal uptake.^21,27,28,51,52^ The utility of STEM-EDXS for characterizing GR sorption behavior was demonstrated in a recent study of As-GR interactions showing that both As­(III) and As­(V) coordinate at GR particle edges through inner-sphere surface complexation reactions, while As­(V) additionally formed parasymplesite precipitates with threadlike morphology.^52^

The aim of the current study was to combine STEM-EDXS analyses with sequential extraction experiments to characterize the distribution and stability of Ni­(II) sorbates on GR at variable sorption times (1 h–3 months). The results provide new insights into the surface properties of GR and its reactivity as a dynamic sorbent of trace metals in reducing systems.

## Materials and Methods

### Anaerobic Conditions and Safety Hazards

To avoid oxidation of GR by O_2(g)_, all experimental procedures were conducted inside an anaerobic glovebox with a 95% *N*_2_(*g*)/5% *H*_2_(*g*) atmosphere. The anaerobic protocols are described in our earlier studies.^27,28^ No unexpected or unusually high safety hazards were encountered.

### Ni­(II)-GR Sorption Samples

The Ni­(II)-sorbed GR samples were prepared with the same methods used previously.^27,28^ Briefly, sulfated GR was synthesized with a coprecipitation method,^53^ as described in Supporting Information Section S1, and aged for 4 days. The GR solids were harvested by vacuum filtration, washed, and resuspended in a 0.05 M Na_2_SO_4_ electrolyte solution amended with 0.5 mM Fe­(II)­SO_4_ and adjusted to pH 7.8 with 10 mM tris­(hydroxymethyl)­aminomethane (TRIS) buffer. The addition of 0.5 mM Fe­(II)­SO_4_ was used to minimize GR dissolution during equilibration, and the experimental pH of 7.8 was selected as it provided conditions representative of the slightly alkaline, reducing environments where GR commonly occurs. The resulting suspension had a GR particle density of 8.2 g L^–1^ (equivalent to a solid-phase Fe concentration of 68 mM, consisting of ∼45 mM Fe­(II) and ∼23 mM Fe­(III)) and was equilibrated for 2 days, during which the Fe­(II)_aq_ concentration increased from 0.5 to 0.98 ± 0.015 mM due to partial dissolution of Fe­(II) from GR as the system approached solid-solution equilibrium, and the suspension attained a stable pH value of 7.98 ± 0.01. The suspension was then spiked with 0.94 mM Ni­(II) through the stepwise addition of 5 aliquots of a 0.1 M NiSO_4(aq)_ stock solution with simultaneous readjustment of pH using 0.1 M NaOH. GR solids were collected by centrifugation after sorption times of 1 h, 1 day, and 3 months. Suspension pH was measured at each sampling time point, with values observed to be 8.0 ± 0.1. The centrifuged solutions were filtered (0.22 μm), acidified, and analyzed for dissolved Ni­(II) and Fe­(II) using inductively coupled plasma-optical emission spectroscopy (ICP-OES), while the solids were collected for analyses by powder X-ray diffraction (XRD), Ni *K*-edge X-ray absorption spectroscopy (XAS), as well as scanning transmission electron microscopy coupled with energy-dispersive X-ray spectroscopy (STEM-EDXS).

### Solid Phase Analyses

The GR samples were analyzed with powder XRD, Ni *K*-edge XAS, and STEM-EDXS. The XRD patterns were recorded on a Bruker D8 diffractometer with Ni-filtered Cu Kα radiation and a LynxEye solid state detector. The XAS data were collected at beamline 12BM of the Advanced Photon Source (APS) and beamline 6BM of the National Synchrotron Light Source II (NSLS-II). Details of the XRD and XAS analyses are described in sections 2–4 of the Supporting Information.

Field emission transmission electron microscopy (FE-TEM) was employed in STEM mode to analyze the morphology and crystal structure of the control and 1-h, 1-day, and 3-months Ni­(II)-GR sorption samples, using a JEM-ARM200F (Cold FEG, JEOL, Japan). A carbon film of a copper grid (C300-25, Ted Pella Inc., USA) was used to hold one drop (∼10 μL) of the diluted mixture (x10) of the control and Ni­(II)-GR suspensions and left to dry overnight under vacuum. The electron beam was focused to a spot size of 0.5 nm. For analysis, the samples were subjected to an acceleration of 200 kV using spherical aberration correctors. Digital images and elemental compositions of the samples were obtained with a Gatan digital camera and energy-dispersive X-ray spectroscopy (EDXS, dual SDD Type), respectively. Analyses were conducted for a surface area of 0.06–0.243 μm^2^, which typically contained multiple GR particles. The samples were carefully monitored for any morphological changes throughout the data collection period. No significant degradation was observed during or after the analysis, as indicated by the intact shape of the GR hexagons under the electron beam during the total observation time (<25 min).

Digital Micrograph software (Gatan, USA) was used to analyze the particle size and *d*-spacing values from the image of selected area electron diffraction (SAED), while the spatial distribution of elements (Fe, Ni, O, Na, and S) within GR particles was assessed using Analysis Station Plus software (JEOL, Japan). EDXS signal intensity profiles of the elements of interest were calculated by integration of the EDXS signals in select areas crossing the GR bulk into the solution.^52^ For each GR sample, 3-to-4 signal profiles were calculated and averaged to generate plots of the EDX signal as a function of scanning distance. This involved analyzing 2–3 GR particles from two STEM images for each sorption sample, and 2 GR particles from one STEM image for the control sample. The details of the data processing protocols used to generate the EDXS signal intensity profiles are described in Section S5 of the Supporting Information.

To minimize the risk of GR dissolution and consequent Ni­(II) desorption and redistribution, the Ni­(II)-GR suspensions used for the STEM-EDXS analyses were prepared with aliquots taken directly from the batch reactors, without filtration and prewashing of the solids with doubly deionized (DDI) water. As a result, precipitates formed from electrolyte components (Na^+^, SO_4_^2–^, Fe^2+^, and Ni^2+^) during sample preparation (see below). These phases were identified as Fe­(OH)_2 (s)_, Ni­(OH)_2 (s)_, and Na_2_SO_4 (s)_ based on combined analyses using STEM-EDXS, SAED, and XRD (see Supporting Information Section S6). To minimize interference, only areas minimally affected by precipitates were selected for the EDXS profile analyses described above.

### Extraction Experiments

Additional experiments were conducted to assess changes in the stability and partitioning of sorbed Ni­(II) over time through stepwise extractions designed to gradually remove Ni­(II) sorbates from the GR solids. Initial attempts employing acids^54–58^ or EDTA^58^ were unsuccessful: the acid extractions resulted in rapid and extensive GR dissolution precluding controlled stepwise extractions, while the EDTA extractions suffered from extensive competition between Fe and Ni for EDTA complexation, as the released Ni­(II) itself formed strong complexes with EDTA in parallel with Fe after each step, preventing accurate characterization of stepwise Ni­(II) release. Successful gradual Ni­(II) extractions were achieved using an electrolyte derived from the GR synthesis suspension, having a distinctly lower pH (pH 7.0) and a much higher [Fe­(II)]_aq_ (46 mM) than the sorption suspensions. To prepare this electrolyte, a 500 mL batch of GR was first synthesized and aged for 4 days, then mixed with 100 mL of a 60 mM TRIS solution adjusted to pH 7.0, and aged for an additional 2 days. The suspension was filtered through a 0.22 μm cellulose membrane to obtain the electrolyte solution, which was in equilibrium with GR. The initial goal of the extractions with this solute was to assess Ni­(II) retention at GR surface sites, with Ni­(II) sorbates mobilized through competitive displacement by H^+^ and Fe^2+^. However, the solute was found to induce dissolution of the Ni-reacted GR solids and therefore mobilized structurally incorporated Ni­(II) as well, whereas no dissolution occurred in Ni-free controls (see results). Ni­(II) substitution thus appears to lower the stability of GR, leading to increased solubility relative to pure GR. As a result, the extractant slightly dissolved Ni-reacted GR, with readsorption of released Ni­(II) suppressed by the relatively high Fe^2+^ and H^+^ levels of the solute.^27^

Ni­(II) extractions were performed on 1-day, 3-weeks, and 3-months aged Ni­(II)-GR sorption samples. A suspension volume of 10 mL was centrifuged, and the supernatant was filtered and acidified. The centrifuged solids were resuspended in 10 mL of extractant solute and equilibrated for 1 day. The suspension then was centrifuged, the supernatant collected, and the solids resuspended in fresh extractant solute. This procedure was repeated for a total of 8 extraction steps. Following the same procedure, an additional set of 20 consecutive extractions was conducted as a control on a GR suspension which had been equilibrated at pH ∼8 and was collected prior to Ni­(II) addition, to assess the response of pure GR to the extractant. The final GR solids from all replenishment cycles were collected and analyzed by XRD to evaluate any mineralogical changes, while the supernatants were analyzed by ICP-OES.

## Results and Discussion

### XRD and EXAFS Results

The XRD patterns of the Ni­(II)-reacted GR samples are compared to that of the GR starting phase in Figure S1. No discernible differences are observed, indicating that the GR bulk mineral structure remained unchanged during the 3-months timespan of the sorption experiments. The Ni *K*-edge EXAFS results are presented in Figure S2 and Table S1. These are consistent with our previous results and demonstrate that sorbed Ni­(II) is incorporated in the octahedral layers of a Ni­(II)_*x*_Fe­(II)_0.67–*x*_Fe­(III)_0.33_(OH)_2_-LDH phase with 0 < *x* ≤ 0.67.^27,28^ The fit results of the three sorption samples are very similar (Figure S2 and Table S1), indicating that the general coordination of sorbed Ni­(II) remained constant over the course of reaction. This does not, however, preclude significant changes in Ni­(II) speciation, since EXAFS cannot resolve the structural differences between Ni­(II)/Fe­(II)–Fe­(III)-LDH phases with compositions in the range between dilute Ni­(II):GR solid solutions, where only a small fraction of structural Fe­(II) is substituted by Ni­(II), to pure Ni­(II)–Fe­(III)-LDH endmembers, where Fe­(II) is completely replaced by Ni­(II). This indicates that Ni­(II) can be structurally incorporated into GR across a continuum of compositions ranging from minor substitution to the development of Ni-rich LDH phases.^27,28^ The STEM-EDXS analyses presented next further evaluate the impacts of sorption time on Ni­(II)-GR association.

### STEM-EDXS Results

#### Morphology of GR Platelets and Precipitates

STEM images of the GR control and sorption samples are presented in Figure 1, with additional images shown in Figures S6–S9 (Supporting Information Section S7). These demonstrate hexagonal GR platelets with diameters of 50–250 nm, consistent with previous TEM studies of GR-sulfate prepared with the coprecipitation method used here.^48–50^ The platelets stack to form GR particles with thicknesses of 20–50 nm as estimated from EELS measurement,^48,52^ corresponding to ∼18–45 stacked platelets.

**Figure 1:**
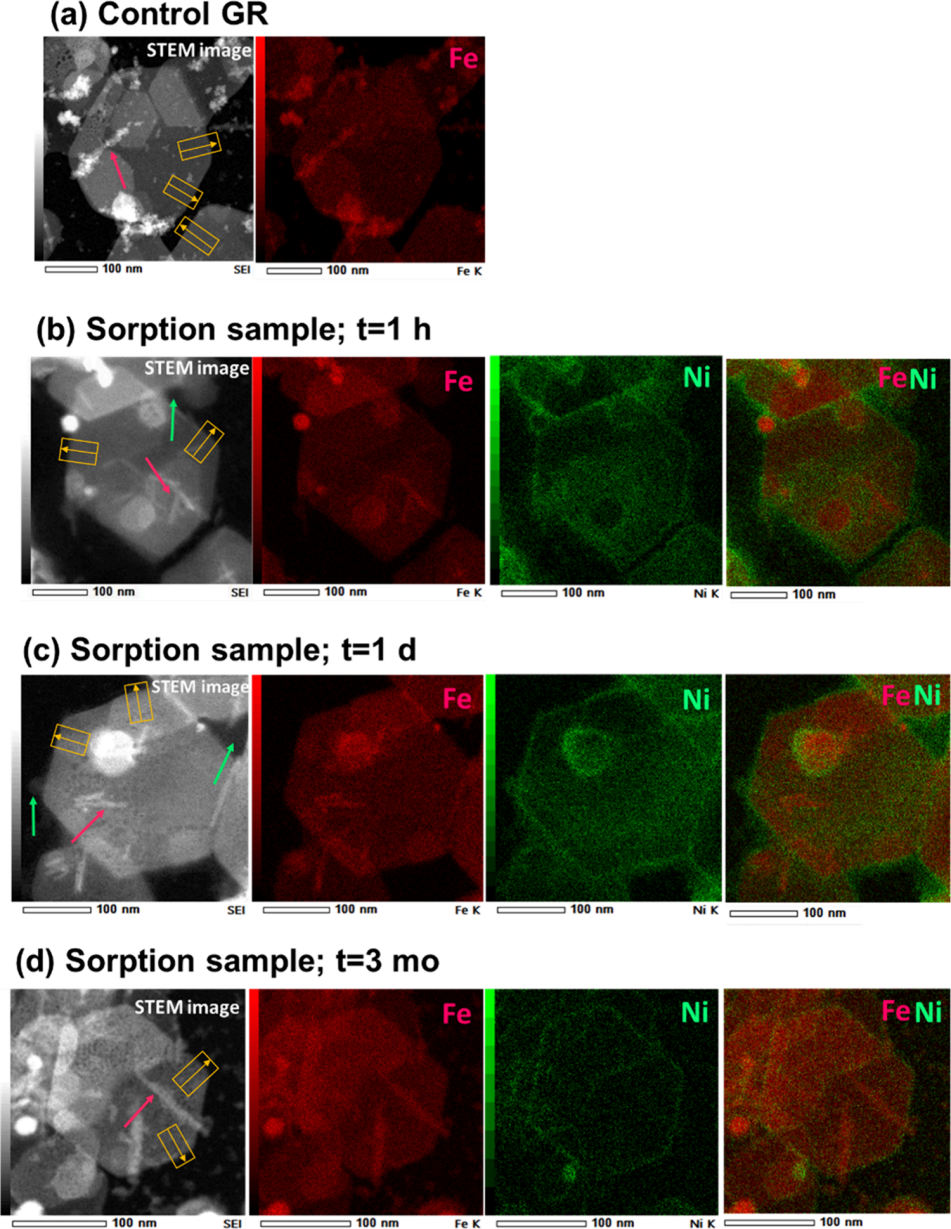
STEM images and the corresponding EDXS maps of Fe (red), Ni (green), and combined Fe and Ni for the (a) GR control sample, and the Ni­(II)-GR sorption samples reacted for (b) 1 h; (c) 1 day; and (d) 3 months. The corresponding EDXS maps of O, Na, and S and the STEM-EDXS results of additional samples are presented in Figures S6–S9. The red and green arrows point to rod-shaped Fe­(OH)_2 (s)_ and diffuse Ni­(OH)_2 (s)_ precipitates, respectively, formed during sample drying. The yellow rectangles represent the areas used for calculating the EDXS signal intensity profiles presented in Figure 2.

Precipitates formed due to the sample drying are observed as well. Needle-shaped Fe­(OH)_2(s)_ particles of 50–100 nm length are present in all samples (red arrows in Figure 1 and Figures S6–S9), and are attributed to the precipitation of Fe­(II)_aq_ from entrained electrolyte as the sample dried out (Supporting Information Section S6). In the 1-h and 1-day sorption samples, diffuse Ni­(OH)_2 (s)_ precipitates are also present (green arrows in Figure 1b,c and Figures S7 and S8), and are similarly attributed to the precipitation of dissolved Ni^2+^ during sample drying. The absence of Ni­(OH)_2 (s)_ in the images of the 3-month sample (Figure 1d and Figure S9) is consistent with the much lower remaining Ni­(II) solution level in this sample compared to the 1-h and 1-day samples because of slow Ni­(II)_aq_ sorption (Table S2). XRD analyses further indicate extensive precipitation of Na_2_SO_4 (s)_ in the samples (Figure S3). Areas with large deposits of Na_2_SO_4 (s)_ were avoided in selecting the regions for imaging and EDXS mapping because of the overlap of Na_2_SO_4 (s)_ particles with GR platelets. For this reason, the STEM images presented in Figure 1 do not display Na_2_SO_4 (s)_ particles despite their abundant presence in the samples.

#### Elemental Distribution Patterns in GR Platelets

EDXS maps showing elemental distributions in GR platelets from the control and sorption samples are presented in Figure 1 (Fe and Ni) and Figures S6–S9 (Fe, Ni, O, S, and Na). As expected, the distributions of Fe, O, and S, which are structural ions of the GR lattice, correlate with the locations of GR particles in the STEM images. The distribution of Na also overlaps with the GR particles, suggesting that Na may be a structural element as well. This is consistent with the structural model of GR proposed by Christiansen et al.,^59^ which contains Na^+^ in the interlayers alongside SO_4_^2–^ and has chemical formula NaFe­(II)_6_Fe­(III)_3_(SO_4_)_2_(OH)_18_·12H_2_O.

The EDXS maps show spatial variability in elemental abundances. Higher Fe and O counts are observed in areas with GR particle overlap and at the location of Fe­(OH)_2 (s)_ precipitates, as expected (Figure 1 and Figures S6–S9). The EDXS maps of the Ni­(II)-sorbed GR samples similarly show elevated Ni counts in areas with Ni­(OH)_2 (s)_ precipitates (Figure 1b,c and Figures S7 and S8). Diffuse patchy enrichment of Ni­(II) on the basal planes of the short-term samples is attributed to Ni­(OH)_2(s)_ precipitation, complemented, possibly, by exchange of Ni^2+^ for Na^+^ in the interlayers. The most notable features in these maps, however, are the bright Ni rims of ∼10 nm lining the particle edges, which are observed in all samples and for all GR particles (Figure 1b–d and Figures S7–S9). This consistent pattern of Ni­(II) accumulation along GR particle edges is not observed for any of the other elements and differs notably from the patchy distribution and diffuse nature of the Ni­(OH)_2 (s)_ precipitates in the 1-h and 1-day sorption samples, indicating a different formation mechanism. The edges of GR platelets are interruptions of the GR mineral lattice and as a result accommodate metal complexation and coprecipitation reactions, unlike the chemically stable and positively charged basal planes.^27,28^ We therefore conclude that the Ni-rich rims observed in the EDXS maps are the result of sorption processes accumulating Ni­(II) along the GR edges.

The EDXS signal intensity profiles allow further assessment of the distribution of Fe, O, S, Na, and Ni in the near-surface regions of the GR crystals.^52^ The results are presented in Figure 2 for the control and the three sorption samples. The structural GR elements (Fe, O, and S) display relatively constant signals in the GR bulk portion of the EDXS profiles, consistent with GR being the primary source of these elements. There are, however, variations between samples in the relative intensities of the elemental signals, most notably for Fe and O. We attribute this to the difference in atomic number between these two elements leading to suppression of the signal of O (Kα = 0.525 keV) relative to Fe (Kα = 6.398 keV) in more thickly stacked particles and in regions with elevated Ni contents. At the particle edges, the signal intensities of Fe, O, S, and Na drop off notably, whereas that of Ni increases (Figure 2b–d) consistent with the Ni-rich rims observed in the EDXS maps. The spatial overlap of Fe and Ni is consistent with the coprecipitation of these two metals to form mixed Ni­(II)_*x*_Fe­(II)_0.67‑x_Fe­(III)_0.33_(OH)_2_-LDH phases as evidenced from the EXAFS results (Figure S2 and Table S1).^27,28^

**Figure 2:**
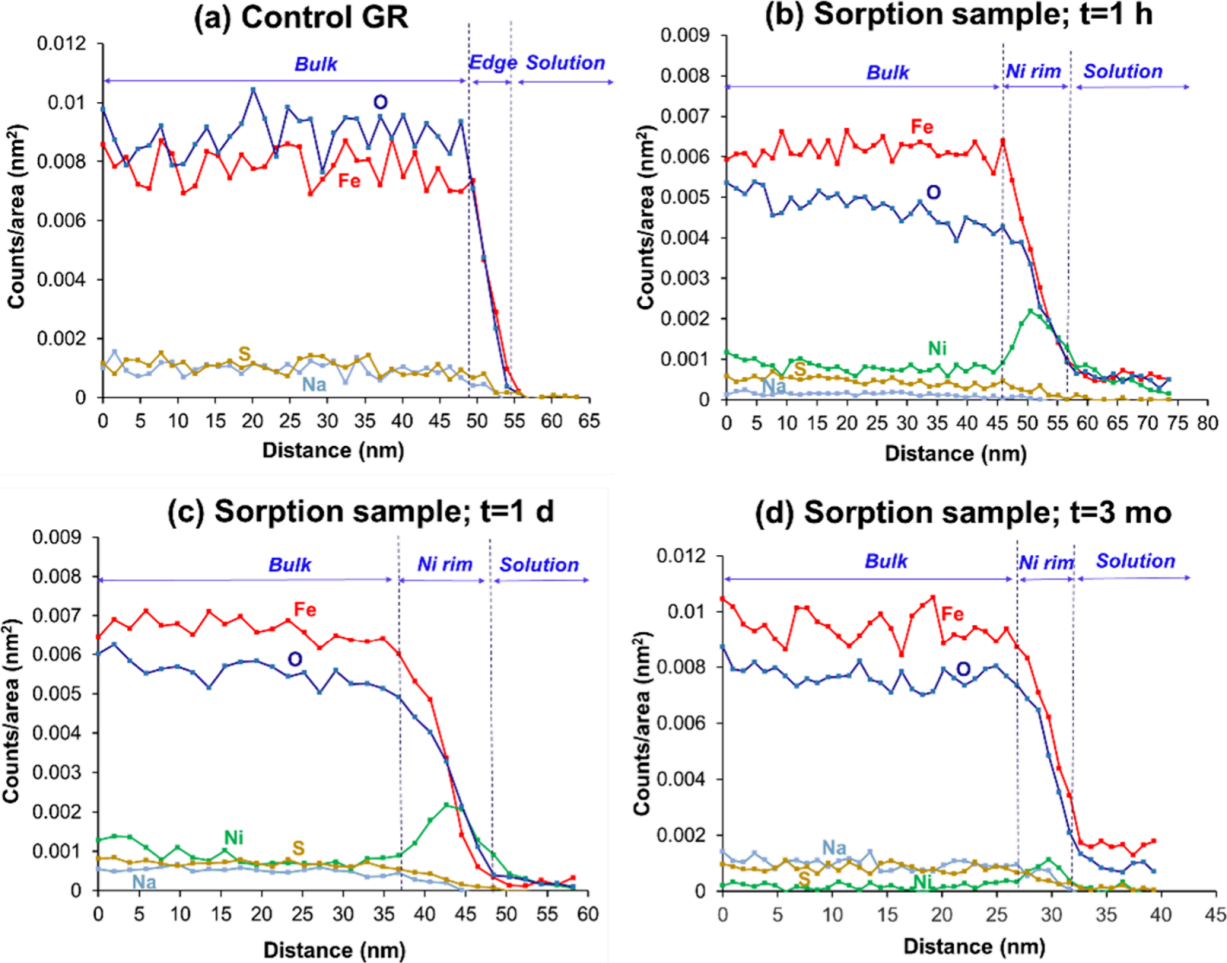
EDXS signal intensity profiles of Ni (green), Fe (red), O (blue), S (orange), and Na (gray) measured for the (a) control GR, and the Ni­(II)-GR sorption samples reacted for (b) 1 h; (c) 1 day; and (d) 3 months. The EDXS profiles are the average of the integrated signals along the arrows drawn in the yellow areas in Figure 1 and Figures S7–S9 for each sample type, starting in the GR bulk and cutting across the particle edges into solution. The individual intensity profiles used for averaging are presented in Figures S10–S13 (Supporting Information Section S8).

An observation of note in the EDXS profiles is that the decrease in the signals of structural Fe, O, Na and S at the particle edges is not a sharp cutoff, but occurs over a 5–10 nm region in both the Ni­(II) sorption samples and the control (Figure 2a–d). This corresponds to 15–30 structural Fe layers, based on the interatomic Fe–Fe distance of ∼3.21 Å in the GR lattice.^60^ These results indicate the presence of a transition zone at the edges where the GR particles are thinner or have a lower density than the bulk. Crystal dissolution during the sorption experiments cannot explain this observation, because the samples were aged and pre-equilibrated with electrolyte containing Fe­(II)_aq_. Some dissolution may have occurred during the dilution step in the sample preparation for TEM. We estimate that this amounts to a maximum of 15% of Fe lost from the near-edge region of the GR particles (see experimental determination in Supporting Information Section S9), whereas the edge region displays a much larger Fe loss of ∼50% relative to the bulk (Figure S14). We infer that this loss of Fe is due to either (*i*) nanoscale offsets in the alignment of stacked GR platelets, causing “smearing” of the Fe levels measured at particle edges, and/or (*ii*) a structurally defective GR crystal lattice in the near-edge region (see structural models in Figure S15). Both scenarios produce an interfacial transition zone with amphoteric sites distributed throughout, implying that metal incorporation may occur across the near-edge region. This is consistent with the accumulation pattern of Ni­(II) in our sorption samples (Figure 1), and further agrees with the results of Perez et al.^52^ who observed that As­(III) and As­(V) form mononuclear inner-sphere adsorption complexes at GR particle edges, where they coordinate at sites in a ∼10 nm thick rim along the surface.

The two options offered to explain the lower density of the GR particle edges may be assessed from the ratio of the Fe and O EDXS signals across the interfacial region of the control sample: the values of Fe:O in the edge are expected to be the same as in the bulk if the platelets were stacked disorderedly, but lower if the platelet edges were structurally defective. The Fe:O intensity profile of the control (Figure S16) suggests that Fe:O remains similar as in the bulk up to at least halfway into the edge region, while values at longer distances are subject to high uncertainty because of low Fe and O counts. This suggests that disordered platelet stacking is at least partially responsible for the lower density of the GR particle edges, although we cannot discount structural defects in the outer portions of the edge region in particular.

#### Ni­(II) Sorption Mechanisms

The possible presence of structurally defective layers at the GR surface demonstrated by the STEM-EDXS results (Figure 2) implies that the sorption of Ni­(II) may proceed at least in part through incorporation into Fe­(II) vacancies in these layers. This vacancy-filling mechanism was not considered in our previous studies,^27,28^ but is consistent with the accumulation of Ni­(II) in the interfacial transition zone (Figures 1 and 2), the rapid formation of mixed Fe­(II)/Ni­(II)–Fe­(III)-LDH observed by EXAFS,^27,28^ and the lack of Fe­(II) release to solution during Ni­(II) sorption through Fe­(II)_s_-Ni­(II)_aq_ cation exchange.^27,28^ We argue that this process may complement the coupled Ni­(II)–Fe­(II) exchange and coprecipitation reactions proposed for the early sorption stages, but does not contribute to slow long-term Ni­(II) sorption.

Constraints on the capacity and dynamics of vacancy filling as a potential mechanism of Ni­(II) sorption are provided by the Fe­(II)-GR sorption experiments of our previous study.^27^ The experiments involved spiking of a pre-equilibrated GR suspension identical to those used here with 1.0 mM Fe­(II), and subsequent monitoring of Fe­(II)_aq_ levels over the course of 1 week. GR was observed to remove 0.3 mM of added Fe­(II), with sorption stabilizing after 2 days.^27^ The removal of excess aqueous Fe­(II) from the pre-equilibrated solution supports the presence of Fe­(II) vacancies capable of Me­(II) uptake, and the relatively quick stabilization of Fe­(II) sorption suggests that the sites are readily accessible, consistent with their location at crystal edges. Since Fe­(II) is a structural cation and a perfect match for the GR lattice, it is reasonable to consider that the amount of Fe­(II) sorbed is the upper limit of Ni­(II) removal through vacancy filling in the current experiments. Ni­(II) sorption during the first 2 days of reaction leads to the removal of 0.5 mM Ni­(II)_aq_,^27,28^ which well exceeds the removal of Fe­(II)_aq_ (0.3 mM). This indicates that, while vacancy filling may be a significant mechanism of Ni­(II)_aq_ uptake in the early sorption stages, it cannot solely account for the amount of Ni­(II) sorbed during this time but is accompanied by Ni­(II)–Fe­(II) coprecipitation.

STEM-EDXS evidence for dissolution-reprecipitation during the initial sorption stages is observed from comparison of the 1-h and 1-day samples to the control (Figure 2a–c). The EDXS signal intensity profiles not only show colocation of sorbed Ni with Fe in the edge region as expected for coprecipitation, but also indicate that the interfacial transition zones of the sorption samples are considerably wider than in the control (∼10 nm versus ∼5 nm; Figure 2a–c). This cannot be explained by mere diffusion and coordination of Ni­(II) sorbates in Fe­(II) vacancy sites of the defective surface layers. Instead, the results point to partial GR dissolution coupled with Fe­(II)–Ni­(II) coprecipitation to form the Ni­(II)-rich rims along the crystal edges.

#### Long-Term Changes in Ni Distribution

Further inspection of the STEM-EDXS data reveals evidence for redistribution of sorbed Ni­(II) during the 3-months timespan of the experiment. The STEM images of the 3-month sample show that the GR platelets have the same hexagonal morphology as the 1-h and 1-day sorption samples and control (Figure 1 and Figures S6–S9), in agreement with the XRD results showing that the sorbent remained intact (Figure S1). However, the EDXS signal intensity profiles of the 3-month sample (Figure 2d) display a notably reduced Ni signal across the particle edges compared to the 1-h and 1-day samples (Figure 2b,c). In addition, the interfacial region with elevated Ni­(II) and declining levels of structural elements (Fe, O, Na, and S) spans merely ∼5 nm in the 3-month sample (Figure 2d) compared to ∼10 nm in the 1-h and 1-day samples (Figure 2b,c). These observations align with the changes in the EDXS signal of Ni relative to Fe in the edge (Table S3), showing a considerable reduction in Ni:Fe in the 3-month sample (0.18) compared to the 1-h (0.59) and 1-day (0.64) samples. These EDXS results indicate that the Ni-rich rims of the 3-month sample are thinner and have a lower Ni content than those of the short-term sorption samples. This is remarkable in that total Ni­(II) sorption approximately doubles between 1 day and 3 months of reaction (Table S2). It therefore appears that sorbed Ni­(II) does not simply accumulate at GR particle edges, but rather is redistributed during aging, undergoing partial transfer from the crystal edges into the bulk. This apparent redistribution of Ni­(II) sorbates is assessed further based on the extraction results presented in the next section.

### Extraction Results

Figure 3 presents the results from the extraction experiments, plotting the fractions of Ni­(II) and Fe­(II) remaining in the solid phase over the course of 8 extraction cycles; the corresponding Ni­(II)_aq_ and Fe­(II)_aq_ concentrations are plotted in Figure S17. The cumulative concentrations of Fe­(II) released during extraction are ∼2 mM in the 1-day sample and ∼9 mM in the 3-week and 3-month samples (Figure S17), which corresponds to ∼5 and ∼20%, respectively, of structural Fe­(II)(s) in the initial GR sorbent. The fate of Fe­(III)(s) released during GR extraction is not entirely clear. The XRD patterns of the extracted 1-day and 3-week solids are identical to that of the initial phase (Figure S18), suggesting that released Fe­(III) precipitates as a poorly- or nanocrystalline secondary phase and/or resorbs onto the remaining GR. The extracted 3-month sample displays a small reflection at ∼22 °2θ (Figure S18) indicating minor precipitation of goethite. No other crystalline Fe­(III) phases were detected, although the formation of additional poorly crystalline phases such as ferrihydrite or lepidocrocite below the XRD detection limit cannot be excluded. Reprecipitated Fe­(III) may incorporate Ni­(II) and thus interfere with the aqueous release of extracted Ni­(II). Despite this potential complication, the extraction results provide useful insights into the effects of time on Ni­(II)-GR sorption that complement the STEM-EDXS results, as discussed next.

**Figure 3:**
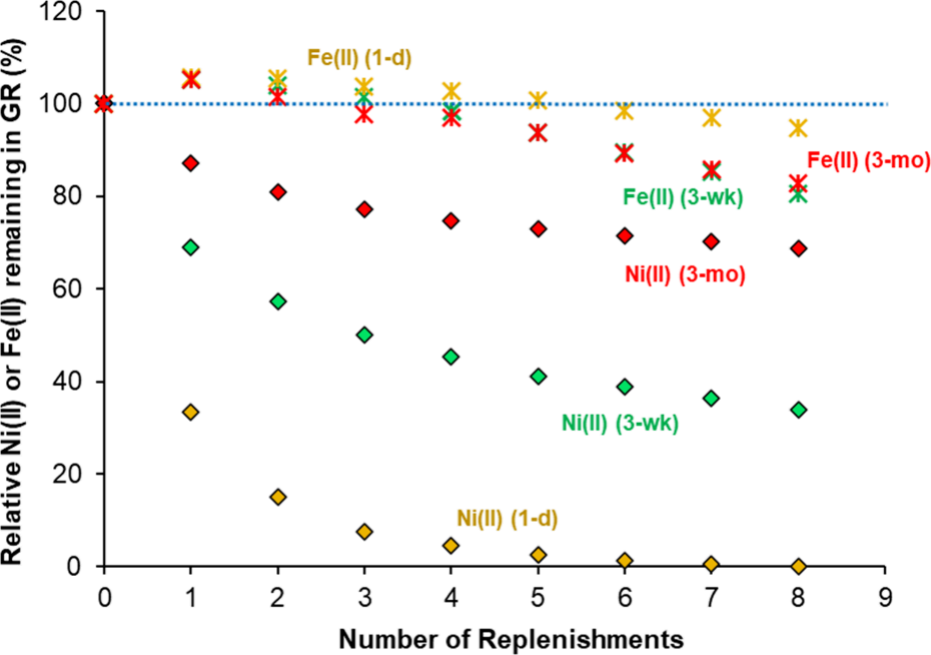
Percentages of Ni­(II) (diamonds) and Fe­(II) (asterisks) remaining on green rust (GR) during sequential extraction of Ni­(II)-GR sorption samples aged for 1 day (yellow), 3 weeks (green), and 3 months (red). The samples were exposed to a total of eight extraction steps. The blue dotted line marks the initial (100%) level. Standard deviations for duplicate samples at all data points were <2%; error bars are smaller than the symbol size and therefore not shown.

Comparison of the Ni­(II) release patterns of the three sorption samples reveal remarkable decreases in the extractability of sorbed Ni­(II) with reaction time. In the 1-day sample, 65% of sorbed Ni­(II) is released during the first extraction step, and essentially all Ni­(II) is desorbed by the sixth step (Figure 3). In the 3-week sample, only 31% of sorbed Ni­(II) is released during the first extraction step, and 34% remains associated with the GR sorbent after the final eighth step. Extractability declines further for the 3-month sample, where merely 10% of sorbed Ni­(II) is mobilized in the first step, and 69% remains on the sorbent after 8 extractions (Figure 3). These changes cannot be explained by differences in the extent of GR dissolution as inferred from Fe­(II) release: the 3-week and 3-month samples display similar Fe­(II) release patterns but distinctly different Ni­(II) extractabilities (Figure 3 and Figure S17), while Fe­(II) release in the 1-day sample is relatively low yet accompanied by complete Ni­(II) desorption (Figure 3 and Figure S17). The results instead point to differences in Ni­(II)-GR association that support the Ni­(II) accumulation and redistribution processes proposed from the STEM observations.

Accumulation of Ni­(II) in a vacancy-rich surface layer is consistent with the changes in [Ni­(II)]_aq_ and [Fe­(II)]_aq_ of the extractant solutions during the first extraction step. For all three samples and the control, Fe­(II)_aq_ levels drop by ∼3 mM in this step (Figure S19). This indicates net sorption consistent with the coordination of Fe­(II) at vacancy surface sites. The uptake of Fe­(II) is accompanied by release of Ni­(II) (Figure 3 and Figure S17) suggesting competitive displacement of Ni­(II) sorbates by Fe­(II) from surface vacancies. The concentrations of displaced Ni­(II) decrease notably with reaction time, with 0.28 mM, 0.19 mM, and 0.08 mM dissolved Ni­(II) observed in the first-step extractant solutions of the 1-day, 3-week, and 3-month samples, respectively (Figure S17). These results point to declining levels of sorbed Ni­(II) in the surface zone, in agreement with the STEM-EDXS results showing that the Ni-rich rims lining GR particle edges become thinner and less concentrated with time (Figure 2).

The second-through-eighth extraction steps induce slight GR dissolution, as evidenced by small yet consistent increases in dissolved [Fe­(II)] during extraction (∼1.5 mM per step; Figure S19). We attribute this to the destabilizing effect of Ni­(II) substitution, rendering the sorption samples slightly undersaturated with respect to the extractant solution as noted in the materials section. The effect is well illustrated by the results of the 3-week and 3-month samples, which exhibit Fe­(II) dissolution throughout the 8-step extraction series accompanied by steady Ni­(II) release (Figures S17 and S19). Dissolution is much less pronounced in the 1-day sample (Figure 3 and Figure S17). This may be explained by the lower Ni­(II) content of this sample (Table S2) and the extensive removal of Ni­(II) sorbates during the first 2 extraction steps leaving the remaining GR sorbent mostly Ni-free (Figure 3).

The observed GR dissolution rate of ∼1.5 mM Fe­(II) per extraction step in the 3-week and 3-month samples corresponds to an estimated removal of a ∼0.42–2.1 nm thick layer around the GR particle edges (see Supporting Information Section S9 for calculation details). The extractions therefore cut through the 5–10 nm Ni­(II)-rich rims identified by STEM-EDXS (Figure 2) and into the mineral bulk as the GR particles are dissolved in a stepwise fashion. The continued release of Ni­(II) from the 3-week and 3-month samples during all 8 extraction steps indicates that Ni­(II) is present inside the lattice well removed from the particle edges. In contrast, Ni­(II) sorbates in the 1-day sample are predominantly concentrated in the near-edge region and largely extracted during the first two steps (∼85%; Figure 3 and Figure S17). These findings support the gradual migration of Ni­(II) into the GR bulk proposed from the STEM-EDXS data.

The linear relation between Ni­(II) and Fe­(II) release in the later extraction steps of the 3-week and 3-month samples (Figure S20) indicate a rather uniform Ni­(II) dispersion within the mineral portion dissolved during steps 2–8. Linear regression yields slopes of 0.012 and 0.009 for the 3-week and 3-month extractions, respectively (Figure S20). These slopes represent the molar ratios of released Ni­(II) and Fe­(II), and their values agree reasonably well with the Ni­(II)/Fe­(II) ratios calculated based on bulk sample composition (Ni­(II)/Fe­(II) = 0.009 and 0.013 for the 3-week and 3-month samples, respectively; see Supporting Information Section S12 for calculation details).This suggests a mostly homogeneous distribution of Ni­(II) in the crystal bulk underlying the Ni-enriched surface zone, consistent with extensive mixing of Ni­(II) with the GR solid over the experimental time course.

The dynamic redistribution of Ni­(II) sorbates evident from the combined STEM-EDXS and extraction results is consistent with dissolution-reprecipitation of the GR sorbent driven by Fe­(II)-catalyzed recrystallization, a mechanism proposed in our earlier studies to explain continued uptake of Ni­(II)_aq_ in long-term sorption experiments.^27,28^ Such migration from the surface into the mineral bulk has previously been observed for sorbed Ni­(II) during Fe­(II)-activated recrystallization of the ferric-(oxyhydr)­oxides goethite and hematite.^55,56,61^ Our current data provide evidence that incorporation and redistribution processes also readily occur for Ni­(II) sorbates as they interact with GR, a mixed-valent iron phase with a much lower structural Fe­(III) content. Future work will employ Fe radioisotopes to quantify the rates of Fe atom cycling between the aqueous and solid phase in GR suspensions and systematically assess the consequent impacts on trace metal retention.

## Conclusions

The results presented here provide new information on the retention and solubility of Ni­(II) and related divalent trace metals such as Co­(II) and Zn­(II) in the broad range of reducing geochemical environments where GR has been documented, including riparian soils, sediments, aquifers, and drinking water distribution systems. We observe that Ni­(II) initially accumulates in ∼10 nm rims of mixed Ni­(II)/Fe­(II)–Fe­(III)-LDH along GR particle edges during the early sorption stages (hours-days) but migrates into the GR bulk over longer time scales (weeks–months) driven, presumably, by Fe­(II)-catalyzed recrystallization of the GR sorbent. These results suggest that Fe­(II)-GR interactions extensively cycle and exchange Fe and Ni ions between the aqueous and solid phase. This has considerable implications for the stability and solubility of Ni­(II) and related trace metal sorbates in Fe-reducing environments. In systems where GR is a net metal sink, the gradual distribution of metals throughout the crystal bulk implies that they will become less susceptible to desorption over time. Conversely, metal sorbates may be readily remobilized when changes in solution conditions revert the net flux across the GR solid-solution interface,^55,56^ making the mineral a metal source. The controls of pertinent geochemical parameters (e.g., pH, concentration, competing ions) on these dynamic processes require further study.

The extraction experiments indicate that Ni­(II) impurities lower the stability of GR, which, in turn, implies that they may also modify its reactivity. This agrees with recent studies which have demonstrated that trace metal sorbates [including Ni­(II)] promote the transformation of GR to Fe­(III)-oxides during heating^54^ and exposure to oxygen^62,63^ and may enhance the reductive dechlorination of chlorinated hydrocarbons by GR.^64^ These impacts are of importance to the stability and reactivity of naturally occurring GR minerals, which commonly contain significant levels of metal impurities.^8,62,65,66^

Our results identify a nanoscale transition zone along GR particle edges with a density lower than the bulk, indicating a structure different than predicted from a “hard wall” model of the GR edge surface. We infer that this interfacial region, which spans ∼15–30 Fe atom layers, results from near-surface defects and/or disordered platelet stacking, producing a surface zone with both amphoteric and fixed-charge sites distributed throughout (Figure S15). The initial formation of Ni­(II)-rich rims may be mediated at least in part by coordination into vacancy sites of this surface layer, a mechanism that was not previously identified with bulk EXAFS analyses. The properties of this interfacial region likely control the capacity and affinity of GR to form inner-sphere adsorption complexes with e.g. phosphate, arsenate, and selenite,^52,67–69^ and to engage in redox reactions.^21,51,65^ Further work using e.g. high-resolution TEM (HRTEM) and electron energy loss spectroscopy (EELS)^70,71^ will be needed to assess its structure and composition, its control on the reactive behavior of GR, and the attendant implications for metal­(loid) retention and speciation in reducing environments containing GR as a reactive mineral component.

## Supplementary Material



## Notes

(1) Sulfated GR synthesis method; (2) XRD analyses methods and results; (3) EXAFS methods and results; (4) description of EXAFS data fitting procedures and table with EXAFS fit results; (5) data processing methods used for generating the EDXS signal intensity profiles; (6) STEM-EDXS, SAED and XRD results for identification of precipitates formed during sample drying; (7) additional STEM images and corresponding EDXS elemental maps; (8) individual EDXS signal intensity profiles of the control and sorption samples; (9) experimental estimation of the extent of dissolution of Fe from the near-edge region of GR particles; (10) structural models of the near-surface edge region of GR platelets; (11) Fe:O EDXS intensity profile of the control sample; (12) additional results from the sequential extraction experiments (PDF)
