# Supplementary material for: Nanoscale Observation of Nickel(II) Sequestration by Green Rust Sulfate
Source: ACS Earth Space Chem. 2025 Oct 28;9(11):2651–60. doi: 10.1021/acsearthspacechem.5c00199 (PMC12641539; doi:10.1021/acsearthspacechem.5c00199)
Supplement: Supplementary file 1 [file sp5c00199_si_001.pdf]

ACS Earth and Space Chemistry

Supporting Information

Nanoscale Observation of Nickel (II) Sequestration by Green Rust Sulfate

Khondaker M.N. Alam[1], Junho Han[1, 2], Bojeong Kim[3], Evert J. Elzinga[1*]

[1]Rutgers University
Department of Earth & Environmental Sciences
101 Warren Street
Newark, NJ 07102, U.S.A.

[2]Seoul National University
Environmental Planning Institute
Gwanak-ro 1
Seoul, 08826, The Republic of Korea

[3]Temple University
Department of Earth and Environmental Science
Philadelphia, PA 19122, U.S.A.

* Corresponding author. Phone: +1 973 353 5238; Email: elzinga@newark.rutgers.edu

34 pages, 20 Figures, 3 Tables

1. Synthesis of sulfated green rust (GR)

Sulfated GR was synthesized following the co-precipitation method of Refait et al.[1] In this procedure, a mixed Fe(II)/Fe(III) sulfate solution with a molar ratio of 3:1 was first prepared by dissolving 4.16 g $FeSO_4 \cdot 7H_2O$ (0.015 mol) and 1.00 g $Fe_2(SO_4)_3 \cdot nH_2O$ (0.005 mol) in 54 mL of doubly deionized (DDI) water (conductivity > 18.2 MΩ cm). The resulting solution had an initial pH of ~2. Precipitation of sulfated GR was achieved by titrating the solution to pH ~ 7 with dropwise addition of 76 mL of 0.3 M NaOH, added stoichiometrically with respect to Fe(III). This procedure yielded sulfated GR with the formula $Fe(II)_{2/3}Fe(III)_{1/3}(OH)_2(SO_4)_{1/6}$, while surplus Fe(II) remained in solution.

2. X-ray Diffraction Analyses

The Ni(II)-GR sorption samples were analyzed by X-ray powder diffraction on a Bruker D8 Advance diffractometer using Ni-filtered Cu Kα radiation and a LynxEye XE detector. The sorption solids were mixed with glycerol inside the glovebox. The glycerol slurries were then taken outside the glovebox and smeared as a thin film onto a low-background holder for analysis. Data collection was done over the 2θ range 5-80° with a resolution of 0.02 °2θ and a counting time of 0.5 second, requiring approximately 0.5 h per scan. The results of the analyses are presented in Figure S1.

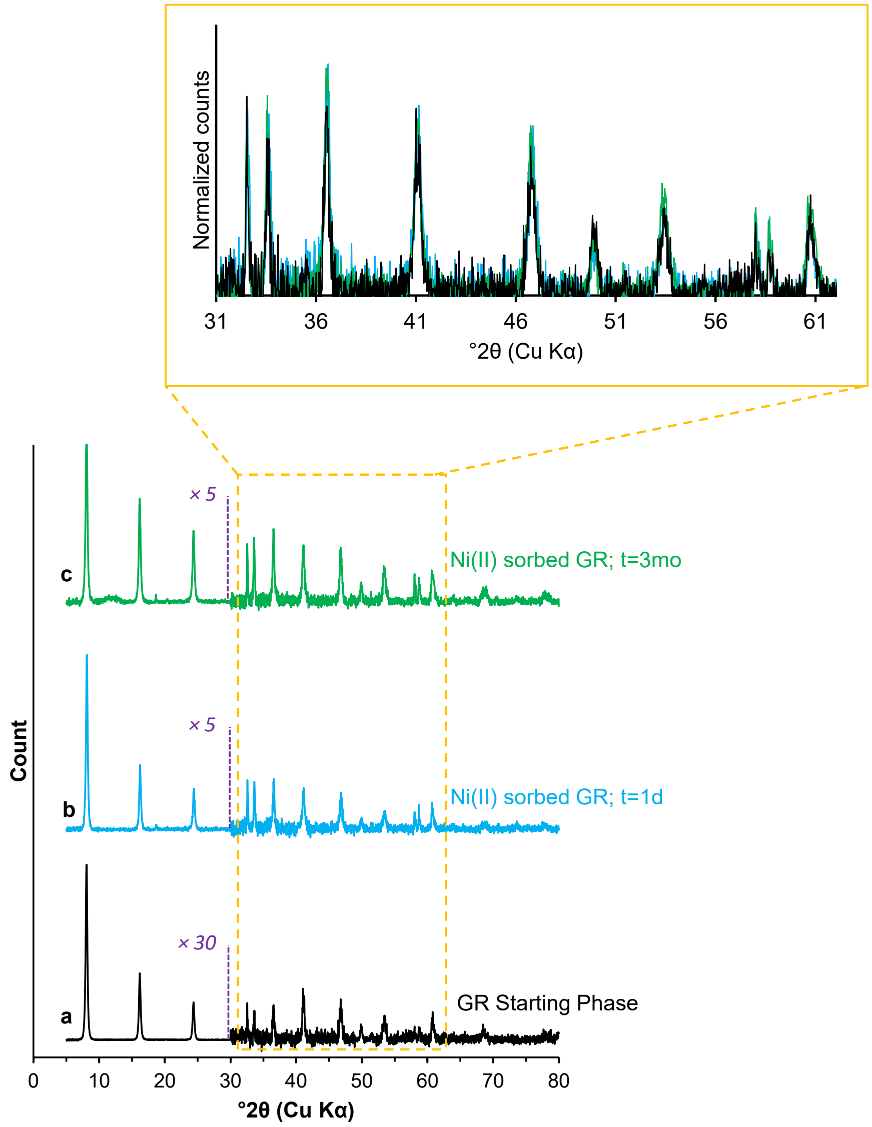

Figure S1. The background-subtracted and normalized XRD patterns of the GR starting phase (a) and Ni(II)-sorbed GR (patterns b, c). The characteristic reflections of GR[2, 3] from the basal planes at low 2θ, and hkl bands in the high-angle sides are present in all samples, indicating that the bulk GR mineralogy has been preserved. No peaks other than those of GR are observed. The strongest peaks at 2θ = ~ 8°, 16°, and 24° correspond to the basal reflections resulting from the layered structure of GR. The regions to the high-angle side of the purple dashed lines were scaled by a factor of 5-30 to enhance the visibility of the hkl bands at 30-80 °2θ. The inset is zoom-ins of the XRD reflections at 31-62 °2θ. All peak positions are within 0.02 °2θ, which was the resolution of the XRD measurement, and there is no evidence for differences in peak width between the patterns of the GR starting phase and sorption samples. This indicates that differences in the relative intensities of the XRD bands between the three patterns are due to effects of preferred orientation.[1] The XRD pattern of the 1-d aged Ni(II)-sorbed GR was obtained from our previous study.[3]

3. X-ray Absorption Spectroscopy Analyses

The Ni(II)-reacted GR solids were sealed as moist samples into lucite sample holders with Kapton tape and transported to the synchrotron facility under anoxic conditions. The XAS data were collected at beamline 12BM of the Advanced Photon Source and beamline 6BM of the National Synchrotron Light Source II. The samples were analyzed in fluorescence mode and at room temperature at the Ni *K*-edge, with up to 15 scans collected per sample. Data processing and fitting are discussed in the section below.

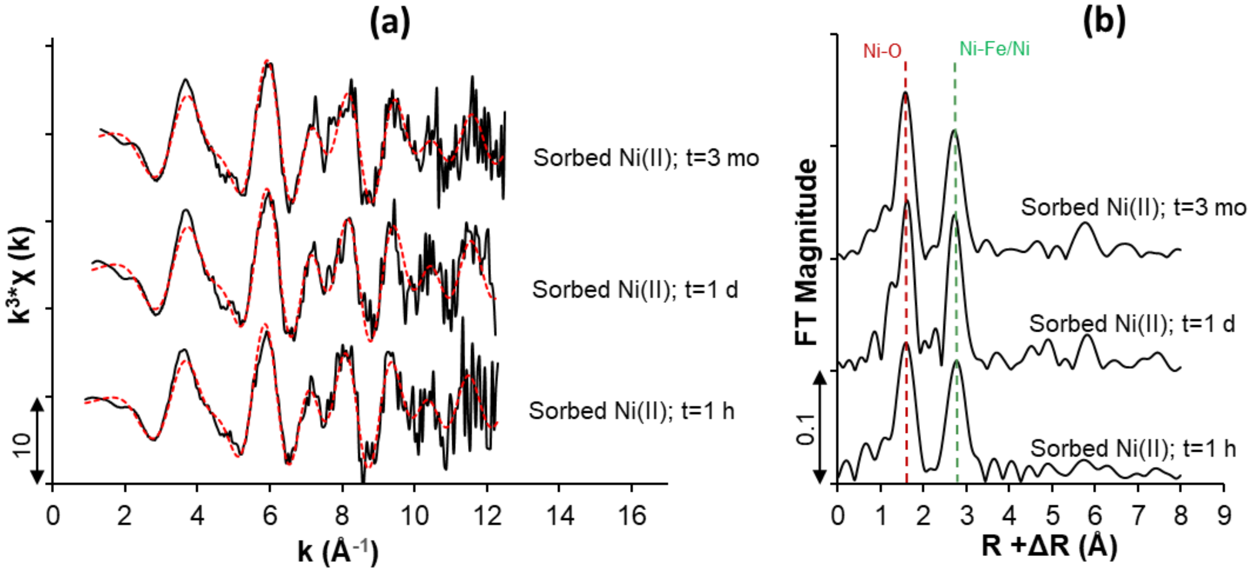

Figure S2. Ni *K*-edge EXAFS data of the sorption samples: (a) k^3-weighted raw (black lines) and fitted (red lines) χ spectra; (b) corresponding Fourier transforms (FTs) of the raw spectra (uncorrected for phase shift). The scaling of the y-axes is indicated by the double-arrow scale bars. The vertical dotted lines in panel (b) locate the first-shell O and second-shell Fe/Ni atomic neighbors surrounding sorbed Ni(II). The 1-h and 1-d aged Ni(II)-GR data were obtained from our previous study.[3] The fit results are summarized in Table S1.

4. EXAFS Shell-by-Shell Fitting

The Ni *K*-edge EXAFS data of the sorption samples were analyzed quantitatively with shell-by-shell fitting using WinXAS 3.1[4] in combination with Athena[5] and Feff7.[6] The structure of β-$Ni(OH)_2$ was used to calculate theoretical scattering paths for each metal, with half of Ni replaced with Fe to obtain theoretical scattering of Fe neighbors. The fit results are summarized in Table S1.

Fitting was performed in R space using the Fourier-transformed k^3-weighted χ spectra to account for the well-defined first and second shells observed in the Fourier transforms (FTs; Figure S2b). The k-ranges used for Fourier transformation were 2.5-11.5 Å^{-1} (1 d and 3 mo samples) and 2.5-10.5 Å^{-1} (1 h sample), while fitting was done over the R range 1.0-3.3 Å. The amplitude reduction factor (S_0^2) was fixed at 0.85, and a single E_0 shift value was allowed to vary during optimization. The samples were fitted with fixed coordination numbers (N) (6 first shell O, and 6 second shell Fe/Ni). The radial distance (R) and EXAFS Debye-Waller factor (σ^2) were optimized during the fitting.

The uncertainties of the fitted R values were previously determined by Elzinga[2] who analyzed Ni(II)-GR sorption and reference samples very similar to those measured here. They were determined based on comparison to crystallopgraphic data of pertinent Ni reference compounds, and amount to ± 0.01 Å for the radial distance of the first shell ($R_{Ni\text{-}O}$), and ± 0.03 Å for the second shell ($R_{Ni\text{-}Fe/Ni}$).

Table S1. EXAFS fit results of the sorption samples.

Sample Type	Shell						$\chi2$ [c]
	Ni-O			Ni-Fe/Ni			
	N[a,b]	R (Å)[a]	σ^2 (Å^2)[a]	N[a,b]	R (Å)[a]	σ^2 (Å^2)[a]	
Ni(II) Sorbed GR-3mo	6	2.05	0.006	6 (Fe)	3.13	0.009	9004
Ni(II) Sorbed GR-1d	6	2.06	0.006	6 (Fe)	3.13	0.007	5235
Ni(II) Sorbed GR-1h	6	2.06	0.006	6 (Fe)	3.14	0.008	2217

[a]N is coordination number, R is radial distance, and σ^2 is the EXAFS Debye-Waller factor, a disorder parameter that accounts for the mean square variation in path length. The standard deviations of the fitted R and σ^2 values were $< \pm 3.1*10^{-4}$ Å and $< \pm 4.4*10^{-5}$ Å^2 in all fits, as determined by WinXAS3.1 [b]Coordination numbers were fixed in all fits; second shell atomic neighbors are indicated in brackets. [c]Goodness-of-fit parameter defined in Ressler.[7] The 1-h and 1-d aged Ni(II)-GR data were obtained from our previous study.[3]

5. Data processing for the EDXS signal intensity profiles

The average EDXS elemental counts in the EDXS signal intensity profiles shown in Figures 2a-d were derived by first measuring and plotting the integrated EDXS signal counts of the individual lines drawn across the areas marked in yellow in Figures S6- S9. The signal counts were then corrected by subtracting the background signals from sample holders. Furthermore, the variations in detector magnifications between duplicate samples were corrected, and differences in X-ray generation and detection efficiency (K-factors) for different elements were accounted for. Subsequently, the processed signal counts were converted to units of counts per nm^2 (counts/nm^2). These normalization steps were essential for enabling meaningful comparisons between sample areas, accounting for differences in particle thickness, and for differences in signal acquisition times between duplicate samples. In addition, a resolution matching process was implemented to ensure consistency in the steps along the x-axes of individual EDXS profiles of the sorption samples (*i.e.,* 1 d Ni(II)-GR, Figure S12), using Essential FTIR software (Operant LLC, USA). This entailed aligning the x-axis points of lower-resolution areas with those of the higher-resolution areas by calculating signal counts of the adjusted steps from the original measurements. Finally, the individual EDXS signal intensity profiles of the respective samples (Figures S10-S13) were aligned with each other and then averaged. This alignment was achieved by using the derivatives of the iron (Fe) signals to identify the corresponding x-axis points at the GR edges where they start to drop, indicative of the beginning of the transition zone where the particles are thinner or less dense than the bulk.

6. Identification of precipitate phases formed during sample drying

To identify the solid phases precipitated during sample drying, a moist and dried 1-h aged Ni(II) sorbed GR samples were mixed with glycerol and analyzed by XRD, with data collected over the 2θ range of 5-80° with a resolution of 0.02 °2θ and a counting time of 4.0 second, taking approximately 4.5 hours per scan. Whereas GR was the only phase observed in the moist sample, $Na_2SO_{4\,(s)}$, $Fe(OH)_{2\,(s)}$, and $Ni(OH)_{2\,(s)}$ were identified as secondary precipitates in the dried sample (Figure S3). In addition, the SAED patterns of the high-resolution TEM images of the needle-shaped particles were analyzed which revealed d-spacing values of 2.8 Å (c) and 2.4 Å (d) in the crystal lattice planes, corresponding to characteristics XRD peaks of $Fe(OH)_{2\,(s)}$, respectively at 31.8° and 37.8° 2θ positions, further confirming the phases as $Fe(OH)_{2\,(s)}$ (Figure S4). Also, the EDXS signal intensity profiles were employed to examine the distribution of Ni within various locations of the STEM images of 1-h Ni(II)-GR sorption sample since the concentration of remaining dissolved Ni(II) was higher in this sample (Table S2). The EDXS profiles presented in figure S5 revealed that the location of the diffused residues exhibited the most abundant signals of Ni where the relative O-to-Ni signal ratio was much higher than the other locations. This suggests a distinct composition in that area, indicating the presence of $Ni(OH)_{2\,(s)}$.

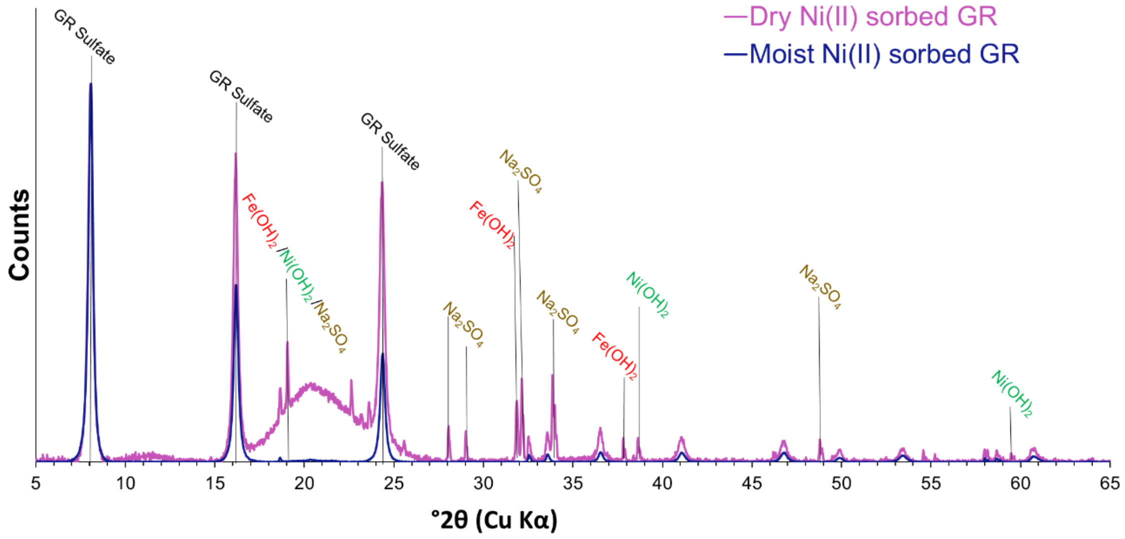

Figure S3. The 4.5-hour scanned, background-subtracted, and normalized XRD patterns of the dry and moist Ni(II)-sorbed GR samples. The dominant diffraction peaks of $Fe(OH)_{2\,(s)}$, $Ni(OH)_{2\,(s)}$, and $Na_2SO_{4\,(s)}$ are observed alongside GR sulfate peaks in the dry sample, but are absent in the moist sample, indicating precipitation of these secondary phases during sample drying.

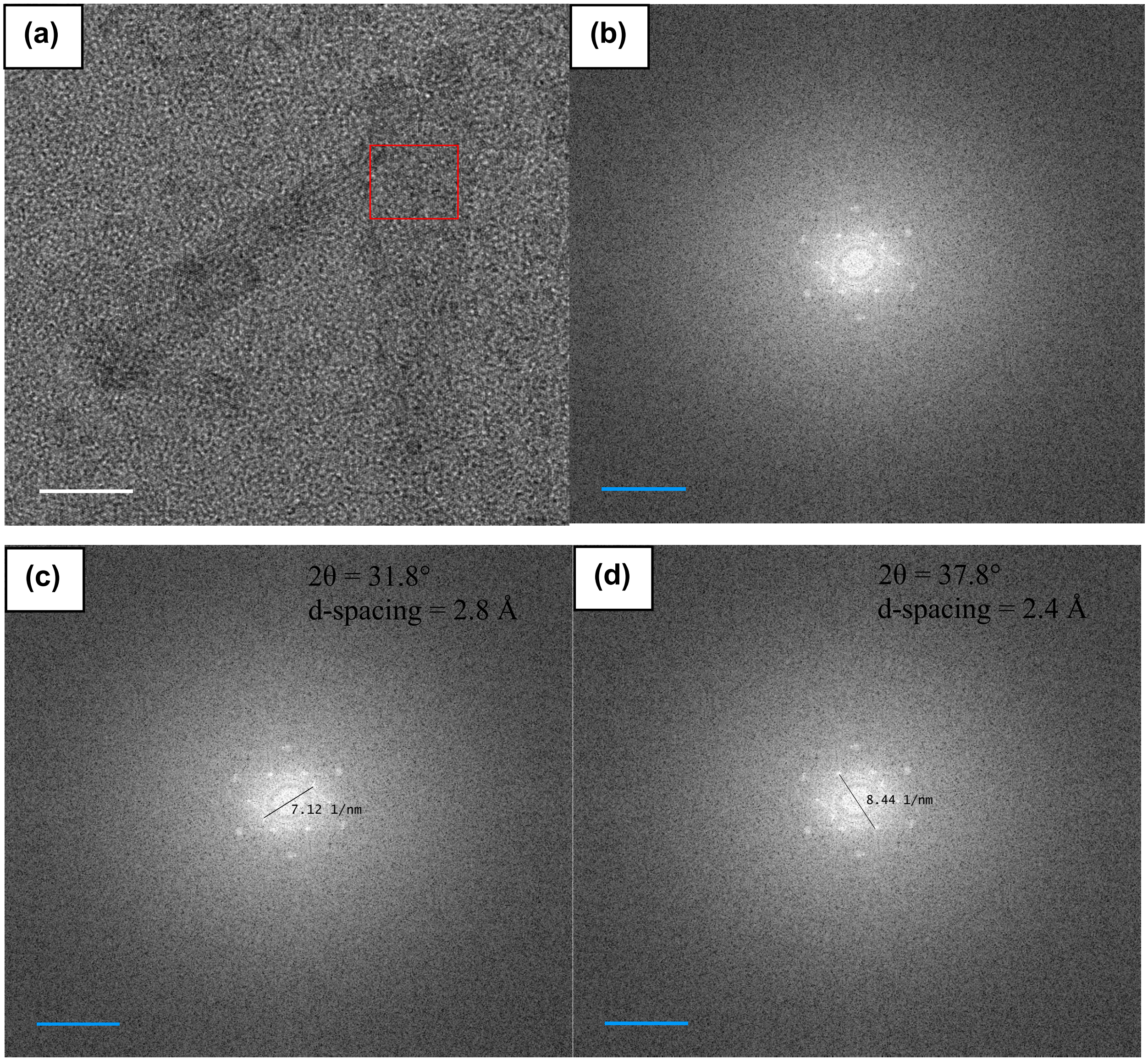

Figure S4: High-resolution TEM (HRTEM) image (a) and the selected area electron diffraction (SAED) pattern of the needle-shaped $Fe(OH)_{2(s)}$ precipitates (b, c, d). The SAED analyses reveal the d-spacing values of 2.8 Å (c) and 2.4 Å (d) in the crystal lattice planes, corresponding to characteristics XRD peaks of $Fe(OH)_2$, respectively at 31.8° and 37.8° 2θ positions.

Table S2. Remaining $Ni(II)_{aq}$ and dissolved Fe(II) concentration in the Ni(II)-GR sorption samples

**Ni(II)-GR Sorption Sample**	**Remaining $Ni(II)_{aq}$ concentration** (Initial $[Ni(II)]_{aq}$ ~ 0.94 mM)	**Dissolved Fe(II) concentration**
1-hour aged Ni(II)-GR	0.62 mM	0.95 mM
1-day aged Ni(II)-GR	0.50 mM	0.93 mM
3-month aged Ni(II)-GR	0.15 mM	0.93 mM

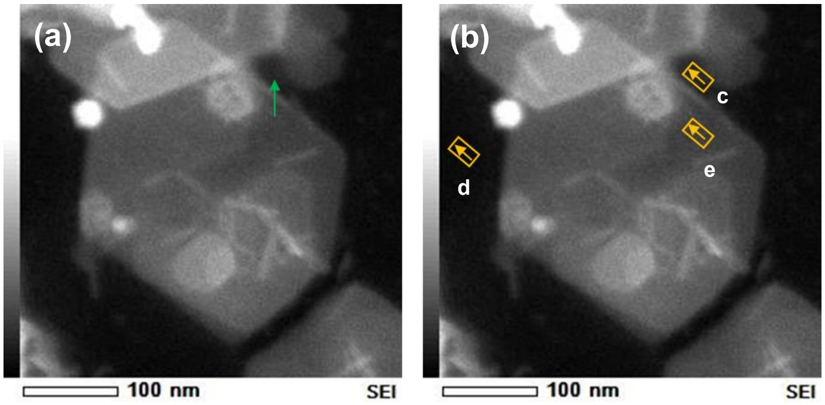

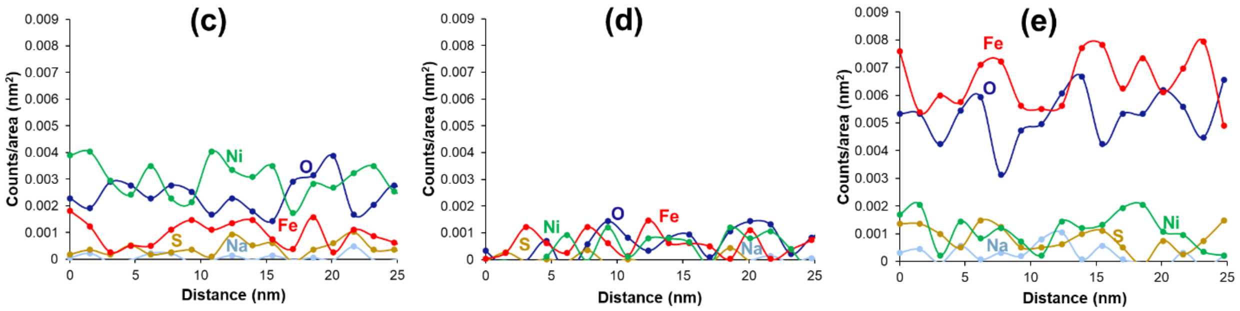

Figure S5: STEM image (a, b) of 1-h Ni(II)-sorbed GR particles. The green arrow shows the secondary diffuse $Ni(OH)_{2\,(s)}$ precipitates. The yellow rectangles labeled c, d, and e represent the areas used to derive the EDXS signal intensity profiles presented in panels (c), (d), and (e), respectively. The yellow arrows in the rectangles indicate the direction of EDXS signal acquisition along the x-axis in the EDXS profiles. The methods of data processing for the EDXS profiles are described below.

7. Additional STEM images and corresponding EDXS elemental maps

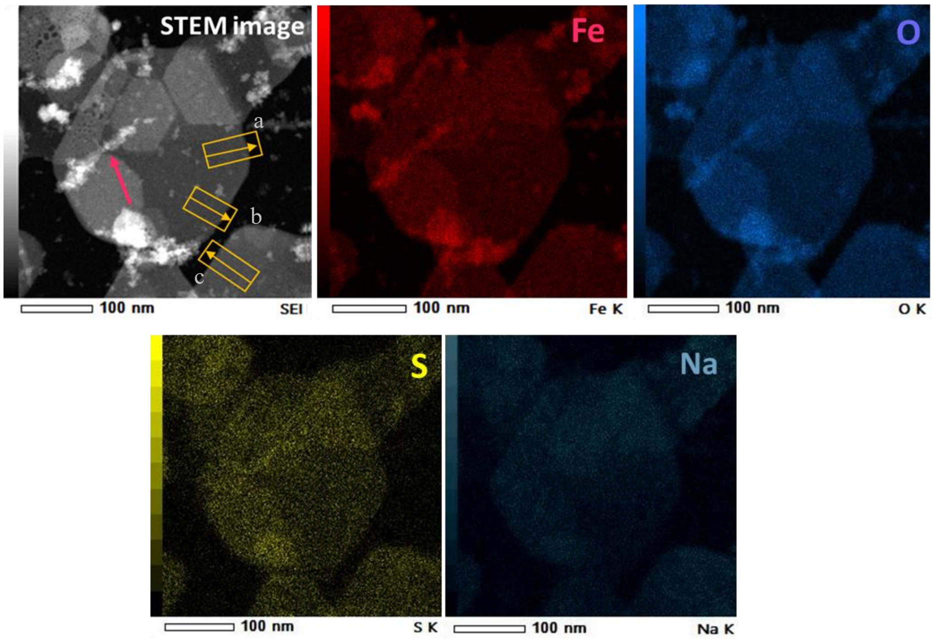

Figure S6. STEM image of control GR and the corresponding EDXS elemental maps: Fe (red); O (blue); S (cyan); and Na (turquoise). The red arrows point to secondary needle-shaped $Fe(OH)_{2\ (s)}$ precipitates. The yellow rectangles represent the selected areas used for the derivation of the EDXS signal intensity profiles in Figure 2a in the manuscript.

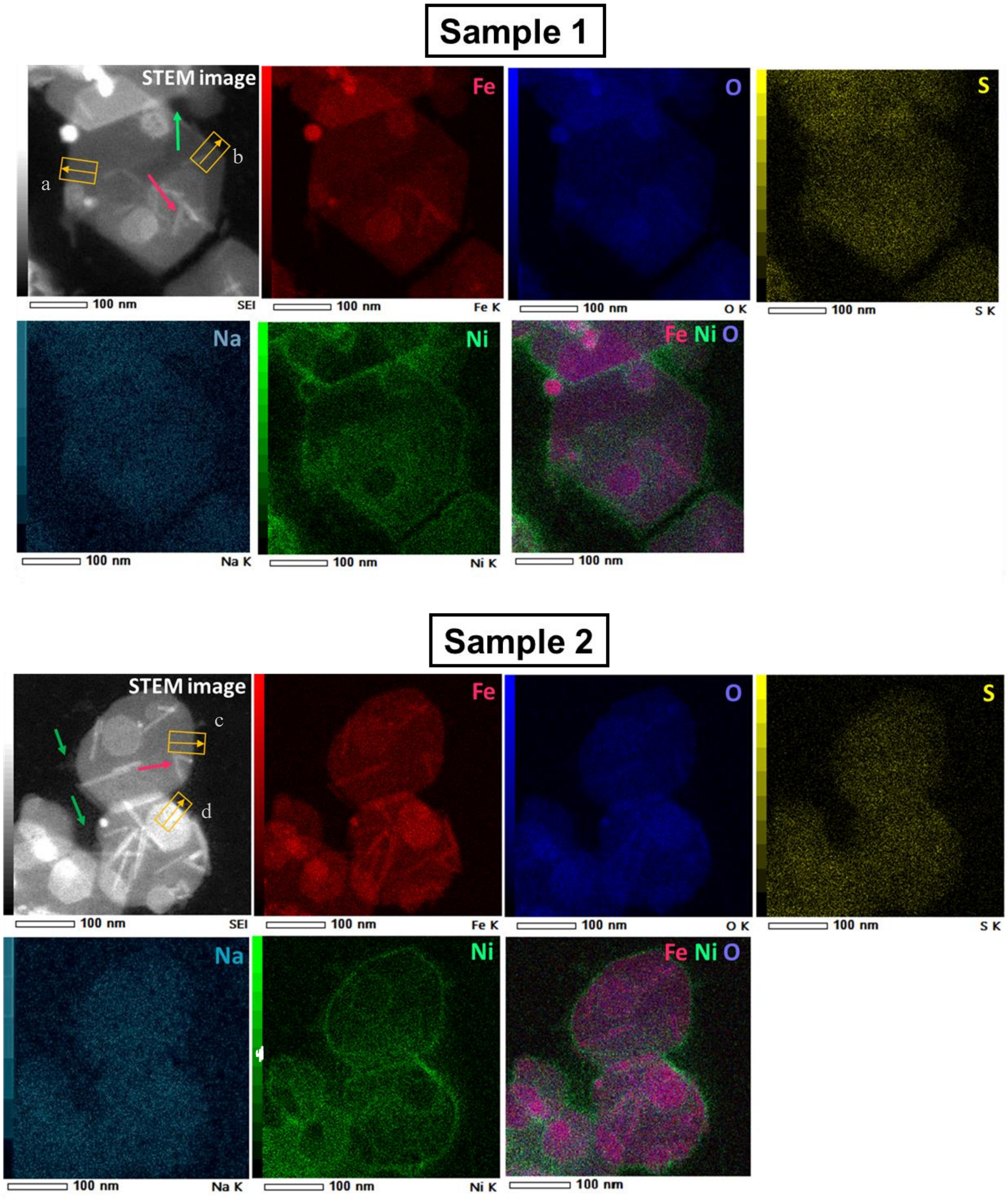

Figure S7. STEM images of 1-hour aged Ni(II)-sorbed GR and the corresponding EDXS elemental maps: Fe (red); O (blue); S (cyan); Na (turquoise); Ni (green); and combined Fe, Ni, and O. The red and green arrows, respectively, indicate the secondary needle-shaped $Fe(OH)_{2\,(s)}$ and diffuse $Ni(OH)_{2\,(s)}$ precipitates. The yellow rectangles are the areas used for the derivation of the EDXS signal intensity profiles in Figure 2b in the manuscript.

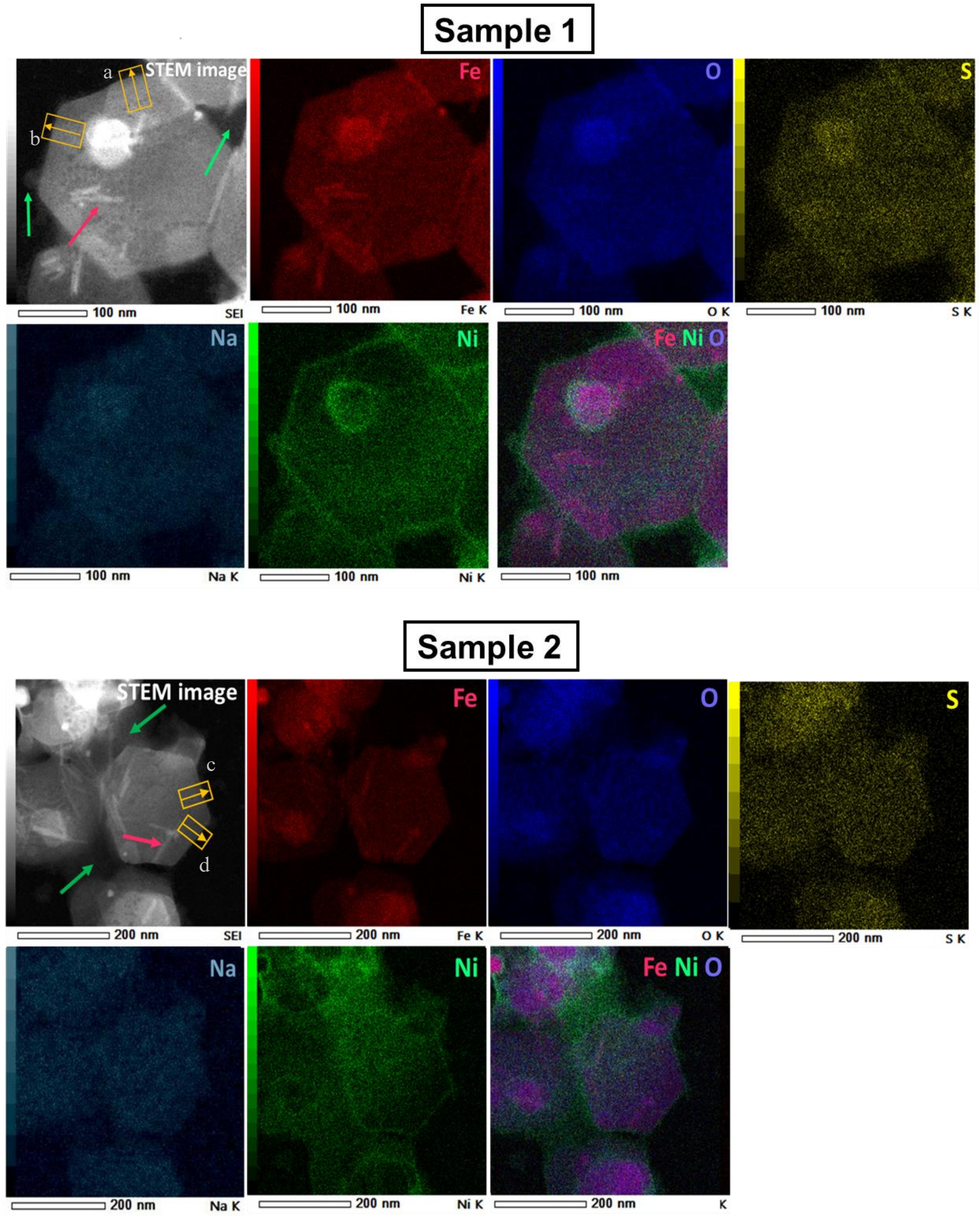

Figure S8. STEM images of 1-day aged Ni(II)-sorbed GR and the corresponding EDXS elemental maps: Fe (red); O (blue); S (cyan); Na (turquoise); Ni (green); and combined Fe, Ni, and O. The red and green arrows, respectively, indicate the secondary needle-shaped $Fe(OH)_{2\,(s)}$ and diffuse $Ni(OH)_{2\,(s)}$ precipitates. The yellow rectangles are the areas used for the derivation of the EDXS signal intensity profiles in Figure 2c in the manuscript.

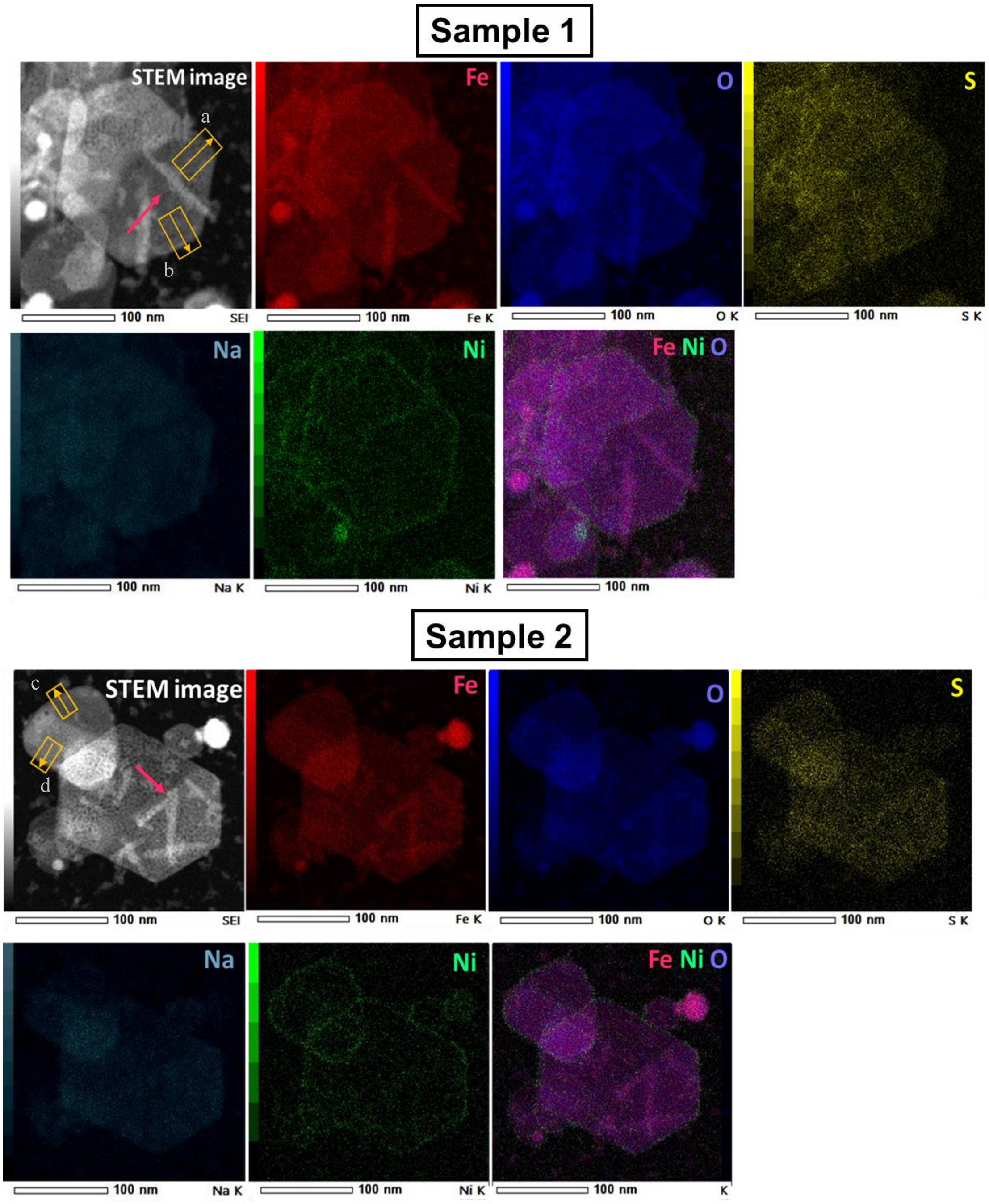

Figure S9. STEM images of 3-month aged Ni(II)-sorbed GR and the corresponding EDXS elemental maps: Fe (red); O (blue); S (cyan); Na (turquoise); Ni (green); and combined Fe, Ni, and O. The red arrows indicate secondary needle-shaped $Fe(OH)_{2\ (s)}$; no $Ni(OH)_{2(s)}$ phases are evident in these images. The yellow rectangles are the areas used for the derivation of the EDXS signal intensity profiles in Figure 2d in the manuscript.

8. Individual EDXS signal intensity profiles of the control and sorption samples

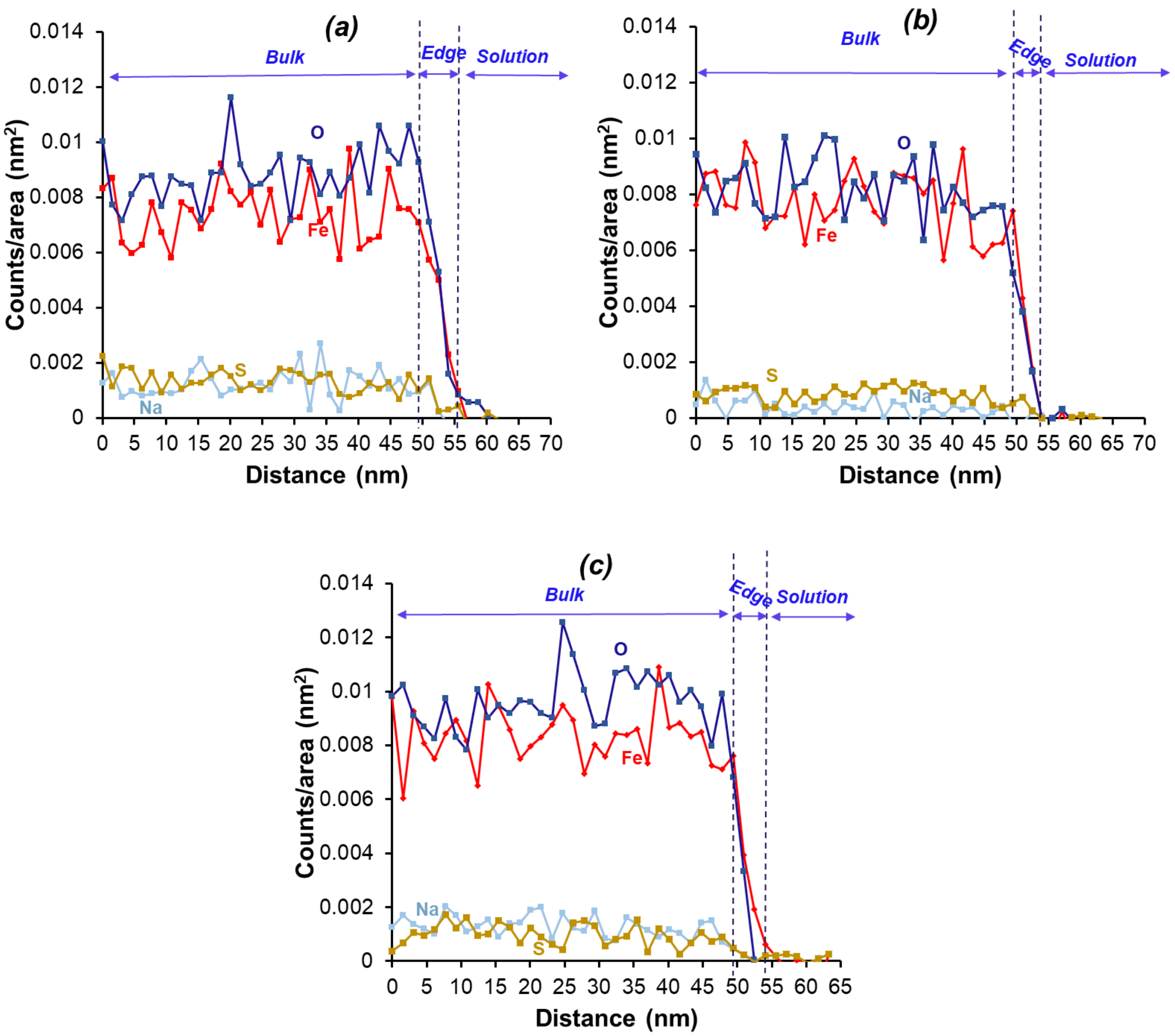

Figure S10. The EDXS signal intensity profiles of elements were obtained from the GR bulk to the solution along the integrated lines drawn across the areas marked in yellow on the STEM image of the control GR on Figure S6. The yellow rectangles labeled a, b, and c correspond to the EDXS profiles (a), (b), and (c), respectively.

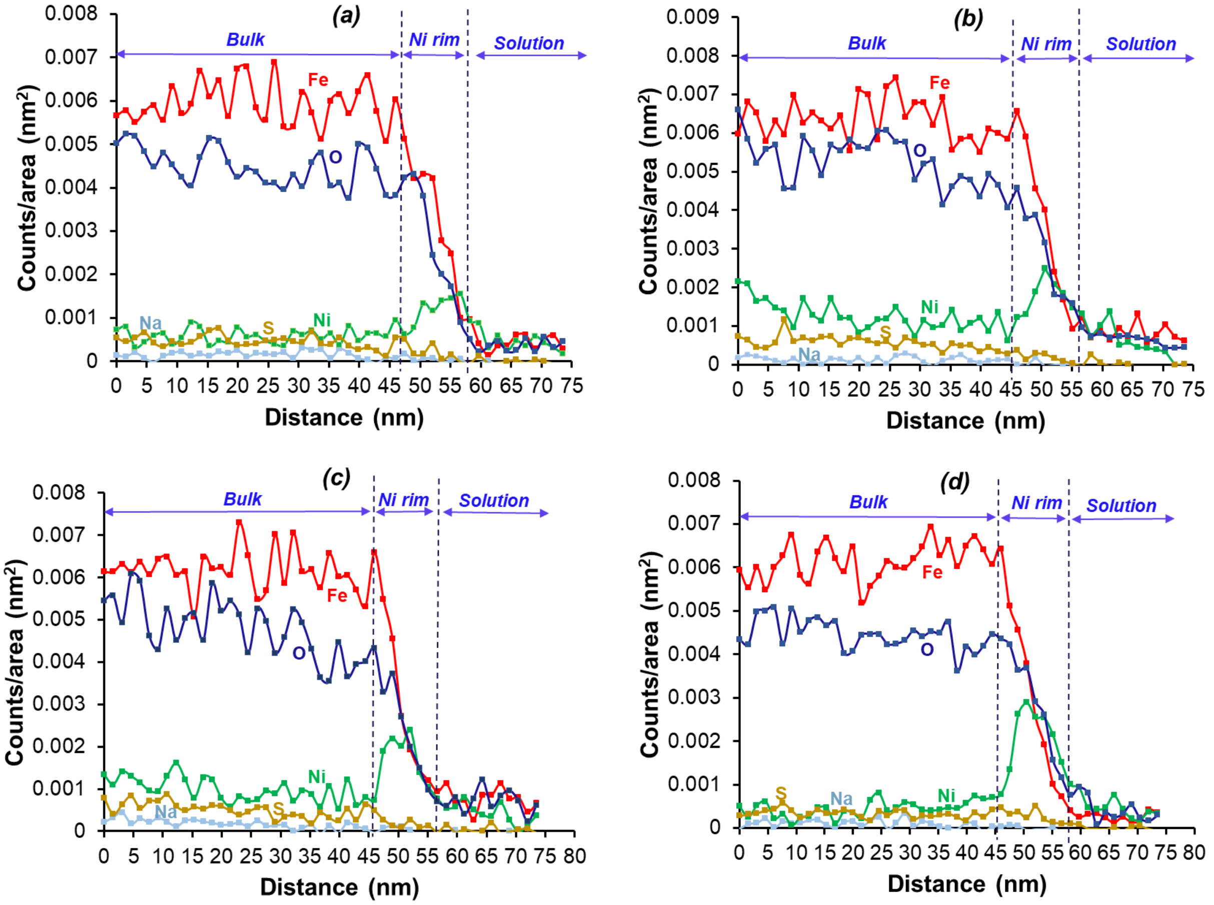

Figure S11. The EDXS signal intensity profiles of elements were obtained from the GR bulk to the solution along the integrated lines drawn across the areas marked in yellow on the STEM images of 1-h Ni(II)-GR on Figure S7. The yellow rectangles labeled a, b, c and d correspond to the EDXS profiles (a), (b), (c) and (d), respectively.

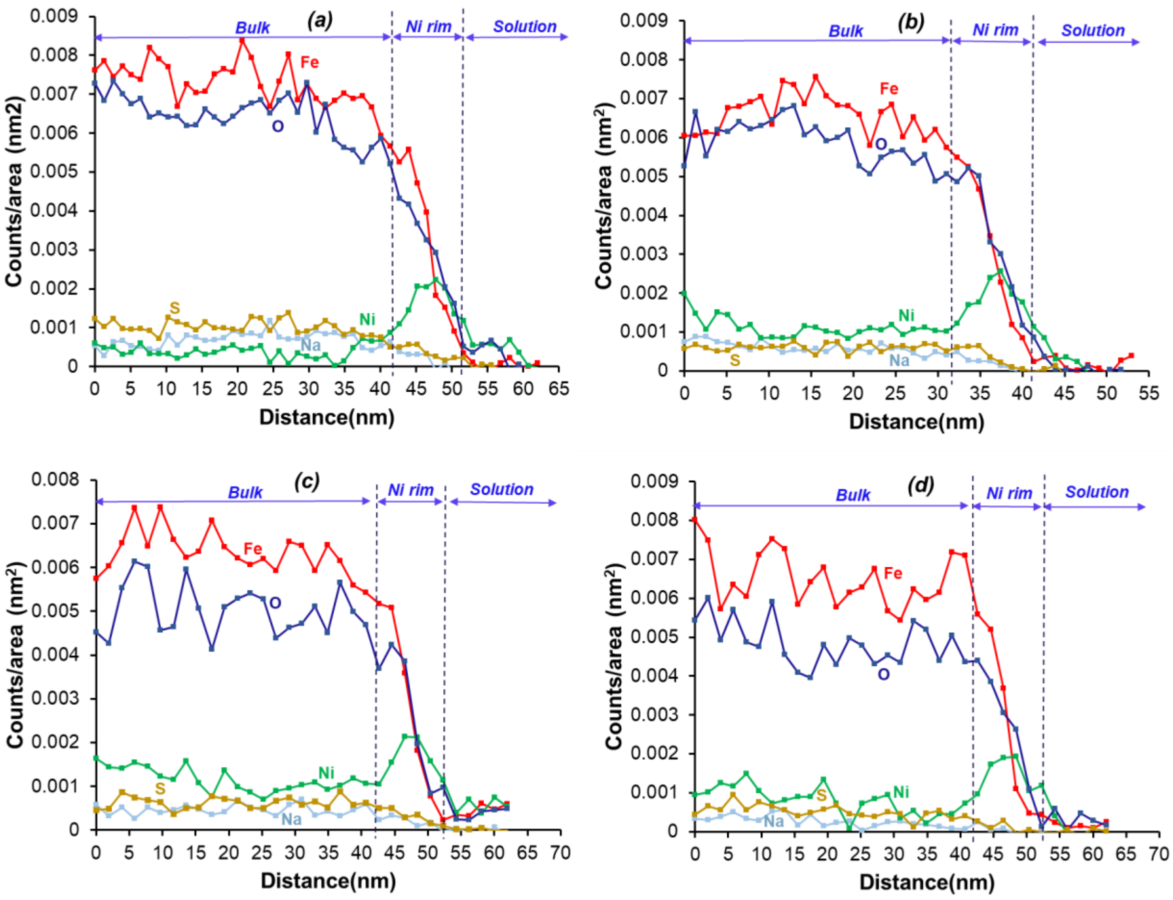

Figure S12. The EDXS signal intensity profiles of elements were obtained from the GR bulk to the solution along the integrated lines drawn across the areas marked in yellow on the STEM images of 1-d Ni(II)-GR on Figure S8. The yellow rectangles labeled a, b, c and d correspond to the EDXS profiles (a), (b), (c) and (d), respectively.

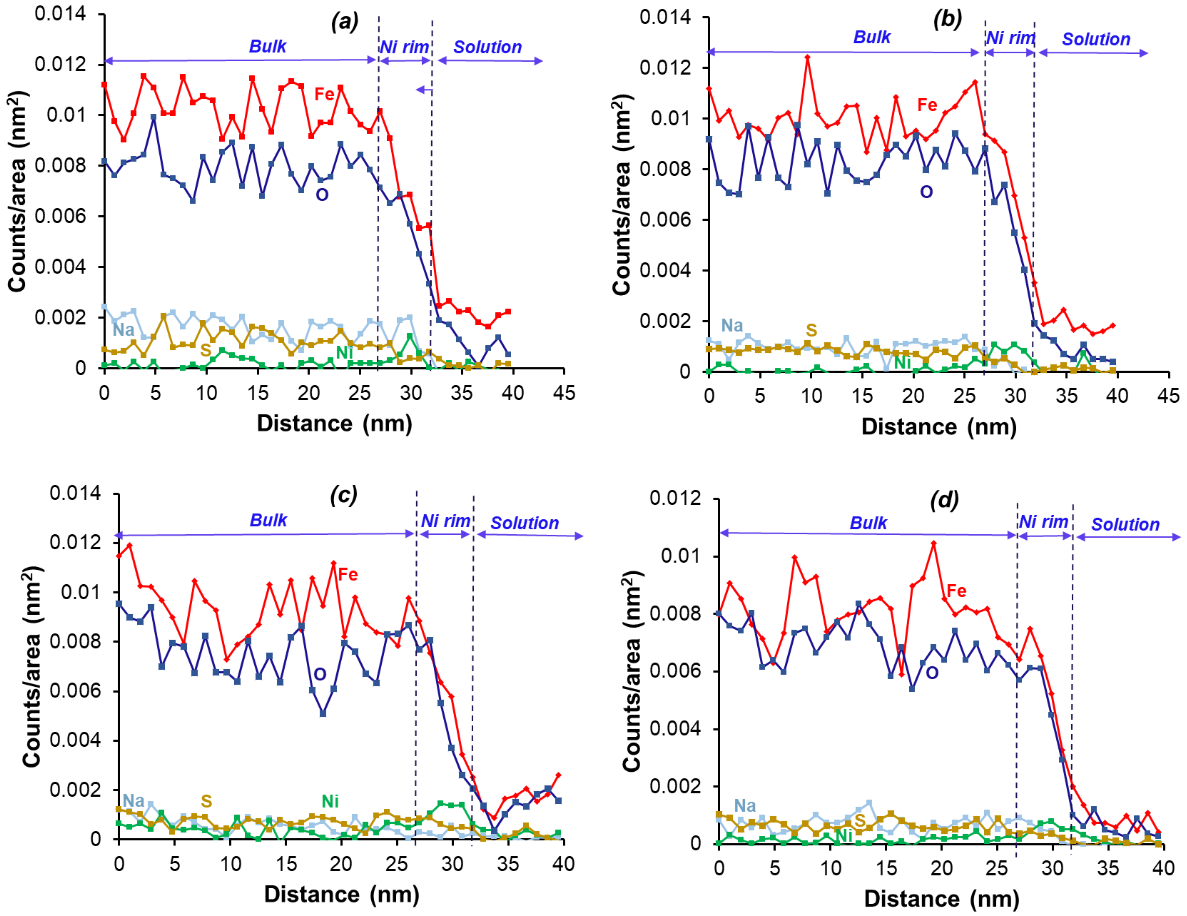

Figure S13. The EDXS signal intensity profiles of elements were obtained from the GR bulk to the solution along the integrated lines drawn across the areas marked in yellow on the STEM images of 3-mo Ni(II)-GR on Figure S9. The yellow rectangles labeled a, b, c and d correspond to the EDXS profiles (a), (b), (c) and (d), respectively.

9. Estimation of extent of GR dissolution during STEM-EDXS sample preparation

To assess the effect of GR dilution during sample preparation for STEM-EDXS analysis, a pre-equilibrated GR suspension was prepared and diluted (×10) with deionized (DI) water, following the same procedure described in the main manuscript. The molarity of total solid-phase Fe was 6.8 mM in the diluted suspension. A ~10 μL droplet of the diluted GR suspension was placed on a TEM grid inside a glovebox and observed to be dried out in less than 30 minutes. The diluted GR bulk suspension was measured to have an initial dissolved Fe(II) concentration of 0.08 mM, which had increased to 0.12 mM after 30 minutes, indicating dissolution of 0.04 mM Fe(II) during this time frame.

The number of Fe atoms contained in a 5 nm zone along GR particles was then estimated using the methods described in the SI of our previous study[1], where we derived equations of the number of Fe edge and bulk atoms of hexagonal GR platelets as a function of particle diameter.[1] Here we employ these equations to estimate the total concentrations of Fe contained in the 5 nm edge region of GR particles for GR particle sizes of 50 and 250 nm, which span the range of sizes observed in our STEM results. We then compare these concentrations to the measured values of Fe dissolved in the dilution experiments to estimate the maximum fraction of Fe mobilized from the edge region.

For a 50 nm particle, the total number of bulk Fe atoms was calculated to be 18,431, while a 45 nm particle contains 14,951 Fe atoms. The difference between these values gives the total Fe atoms located in the 5 nm edge region of the 50 nm particle. The fraction of total Fe in the 5 nm edge region is:

$$(18{,}431 - 14{,}951) / 18{,}431 = 0.1888$$

Therefore, the molarity of Fe (in mM) contained in the 5 nm edge region is:

$$6.8 \times 0.1888 = 1.28 \text{ mM}$$

This compares to 0.04 mM of Fe released during the dilution experiments, which corresponds to the following percentage:

$$0.04 / 1.28 \times 100 = 3.16\%$$

Thus, for 50 nm particles, a maximum of 3.16% of the total Fe in the 5 nm edge region would dissolve.

For a 250 nm particle, the total number of Fe atoms was calculated to be 456,085, and a 245 nm particle contained 438,047 Fe atoms. The fraction of total Fe in the 5 nm edge region:

$$(456{,}085 - 438{,}047) / 456{,}085 = 0.03955$$

Therefore, the molarity of Fe (mM) in the 5 nm edge region is:

$$6.8 \times 0.03955 = 0.27$$

And the percentage of Fe dissolved from this region would be:

$$0.04 / 0.27 \times 100 = 14.8\%.$$

Thus, for 250 nm particles, 14.8% of the total Fe in the 5 nm edge region may be dissolved during the dilution experiments.

Since the actual GR sorbent sample used for the experiments contains a mixture of particle sizes between 50 and 250 nm (Figure 1), we estimate that the fraction of Fe dissolved from the 5 nm edge ranges between 3.16% and 14.8%.

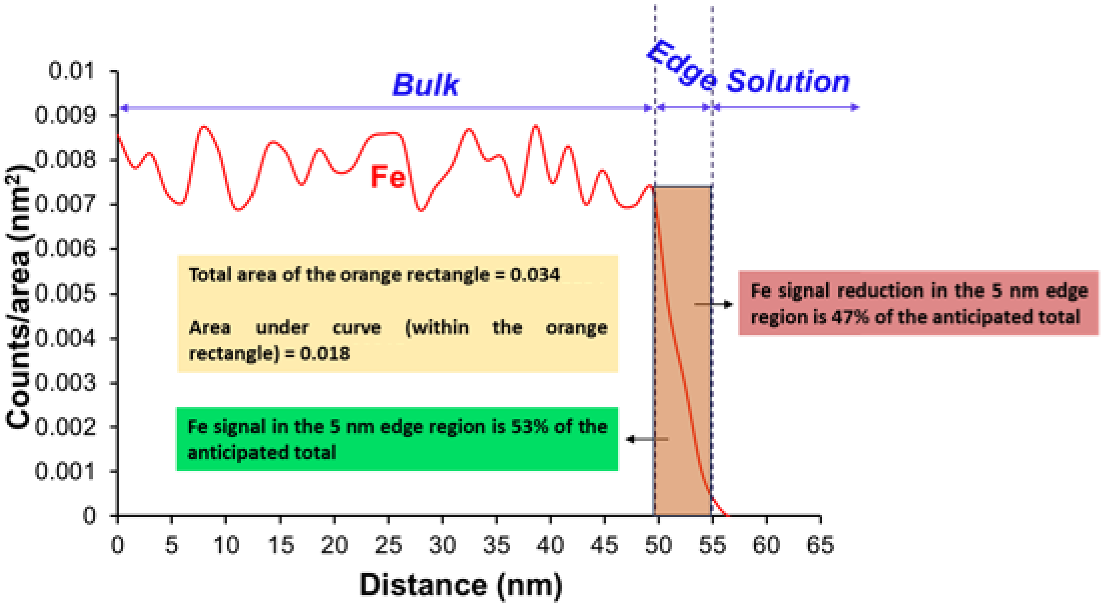

Figure S14. Approximately half of the anticipated total Fe signal of the control GR in the edge region is observed. The area represented by the brown rectangle in the graph corresponds to the total Fe signal in the edge region assuming it has the same Fe content as the bulk. The area under the curve within this rectangle accounts for 53% of the expected total Fe signal, reflecting a 47% decrease in Fe content relative to the bulk.

10. Structural models of the near-surface edge region of GR platelets

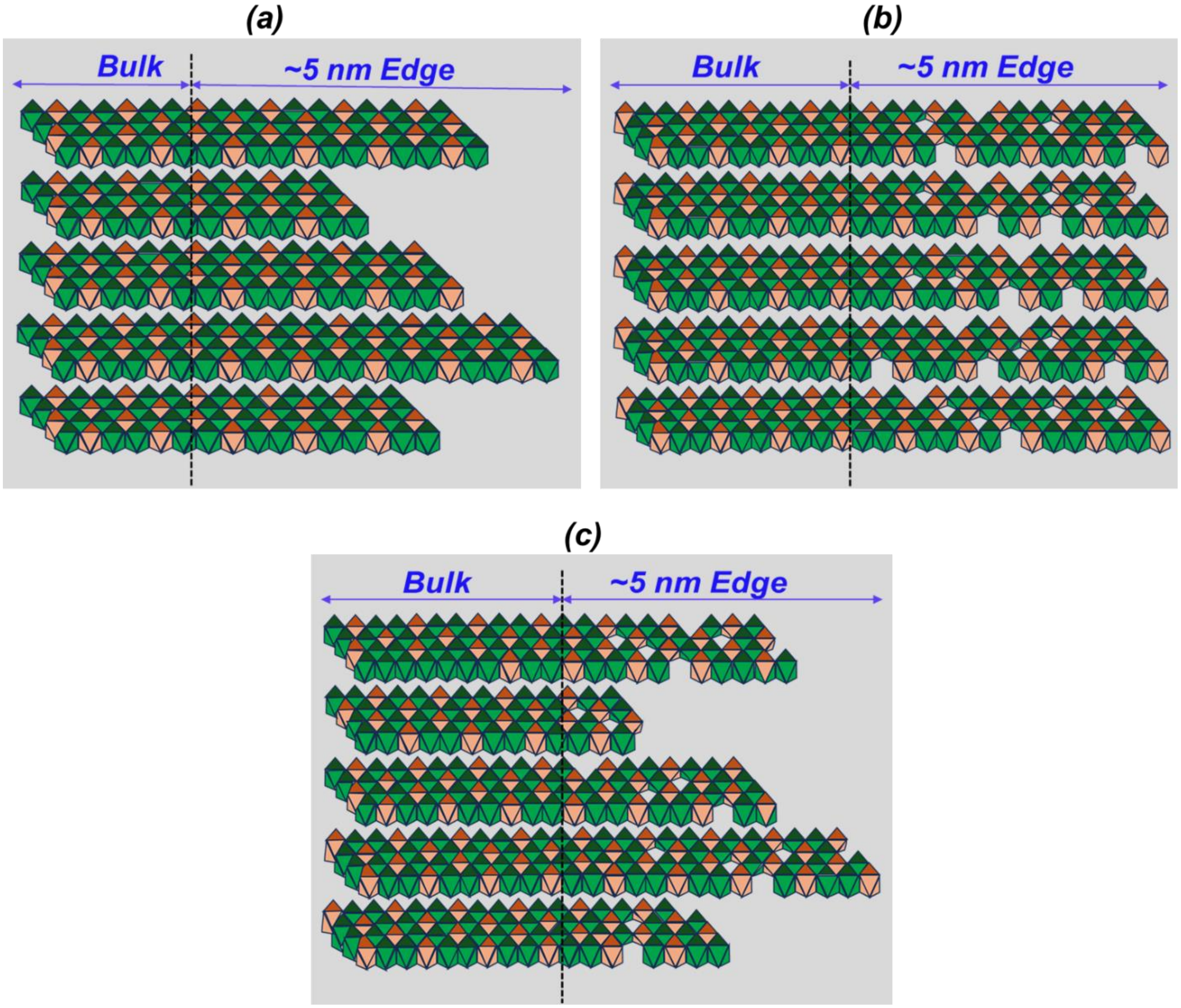

Figure S15. Schematic illustration of potential structural and compositional variations in the near-edge region (~5 nm) of GR particles. Green and brown octahedra represent Fe(II) and Fe(III), respectively. Panel (a) shows nanoscale offsets in the alignment of stacked GR platelets, and panel (b) depicts a structurally defective GR crystal lattice with aligned stacking. Panel (c) illustrates both stacking offsets and lattice defects near the particle edge.

11. The Fe:O EDXS intensity profile of the control sample

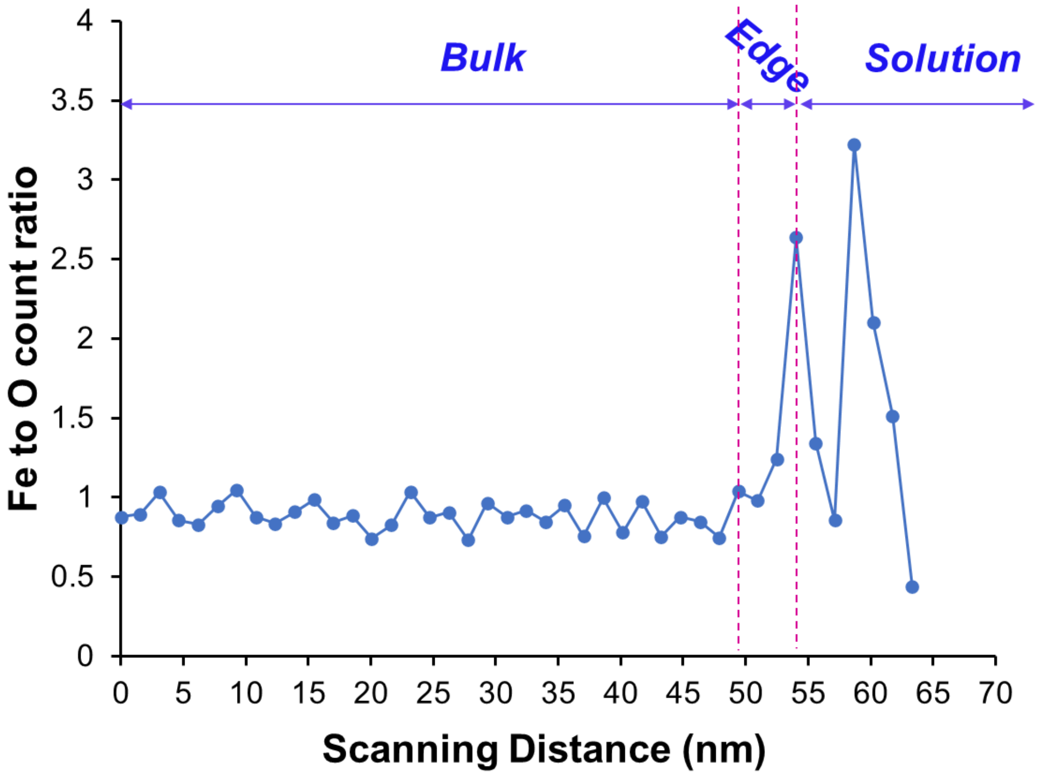

Figure S16. The EDXS signal intensity profile, showing the average Fe to O count ratio in the control sample, from the GR bulk across the surface into the solution along the integrated lines drawn across the areas marked in yellow in Figure 1a.

Table S3. The EDXS signal count ratios of Ni to Fe in GR edges of the sorption samples

Sorption samples	Ni to Fe count ratio at GR edge where the Ni count is highest
1-hour aged Ni(II)-GR	0.59
1-day aged Ni(II)-GR	0.64
3-month aged Ni(II)-GR	0.18

12. Sequential extractions of sorbed Ni(II) from GR

Sequential extractions were performed to evaluate Ni(II) retention in GR samples aged for 1 day, 3 weeks, and 3 months. Figure S17 presents the cumulative release of Ni(II) and Fe(II) from GR over the course of 8 extraction steps. Rapid and extensive Ni(II) release was observed from the 1-day sample, but extractability decreased markedly with aging, as indicated by the much lower cumulative Ni(II) concentrations released from the 3-week and 3-month samples. The cumulative Fe(II) release from these older samples, however, was distinctly higher than for the 1-day sample (Figure S17), indicating more extensive GR dissolution. These differences indicate that Ni(II) becomes less extractable over time and increasingly retained within the GR bulk. XRD patterns of the extracted solids (Figure S18) confirm the structural preservation of GR throughout the extraction process.

Extractions performed on control GR showed much less, if any, Fe(II) release compared to the Ni-GR sorption samples (Figure S19). This indicates that the incorporation of Ni(II) impurities into the GR lattice destabilizes the mineral structure and promotes its dissolution by the extractant.

Fe(II) and Ni(II) release correlations

Linear regressions of cumulative Ni(II) versus Fe(II) release (Figure S18) show strong correlations for the aged samples, indicating that Ni(II) and Fe(II) were released proportionally during continued dissolution. To assess the extent of Ni(II) incorporation into the GR structure, bulk Ni(II)/Fe(II) molar ratios were calculated based on the metal contents measured after the first extraction step. Following the first extraction, the GR solids contained 0.42 mM Ni(II) for

the 3-week sample and 0.57 mM Ni(II) for the 3-month sample. The total Fe concentration in the GR was measured to be 68 mM, with 2/3 of this value (45.33 mM) corresponding to structural Fe(II). The bulk Ni(II)/Fe(II) molar ratios were calculated as:

$$\text{Ni(II)/Fe(II)} = 0.42/45.33 = 0.009 \text{ (3-week sample)}$$

$$\text{Ni(II)/Fe(II)} = 0.57/45.33 = 0.013 \text{ (3-month sample)}$$

These ratios align reasonably well with the slopes derived from the Ni(II)-Fe(II) release regressions (Figure S19), suggesting mostly homogeneous mixing of Ni(II) in the mineral lattice.

Estimated thickness reduction of GR particles during extraction

The thickness of material removed from GR particle edges during each extraction step was estimated based on the observed Fe(II) dissolution rate of ~1.5 mM per step. Given that Fe(II) accounts for 2/3 of the total Fe in GR, this corresponds to a total Fe loss of 2.25 mM per extraction. With an initial total Fe concentration of 68 mM, the fractional Fe loss per step is:

$$\text{Fractional Fe loss} = 2.25 \text{ mM} \div 68 \text{ mM} = 0.0331$$

Because GR particles are hexagonal platelets with uniformly distributed Fe, the particle area, and thus the total Fe content, scales directly with the square of the particle diameter.[1] Consequently, the particle diameter after one extraction step is:

$$d = d_0 \times \sqrt{(1 - 0.0331)} = d_0 \times 0.9833, \text{ where } d_0 \text{ is the initial diameter.}$$

GR dissolution and particle thinning during extraction was evaluated for initial particle sizes of 50 and 250 nm. For d_0 = 50 nm, the post-extraction diameter is 49.165 nm after 1 step, corresponding to a diameter decrease of 0.835 nm and a thickness reduction of 0.42 nm along

each edge. For d_0 = 250 nm, the post-extraction diameter is calculated as 245.825 nm, corresponding to a diameter decrease of 4.175 nm and a thickness reduction of approximately 2.1 nm from each edge. Thus, each extraction step results in the removal of an estimated ~0.42 nm to ~2.1 nm thick layer around the GR particle edges. This corresponds to a total dissolution of ~3-16 nm over the course of the 8-step extraction series.

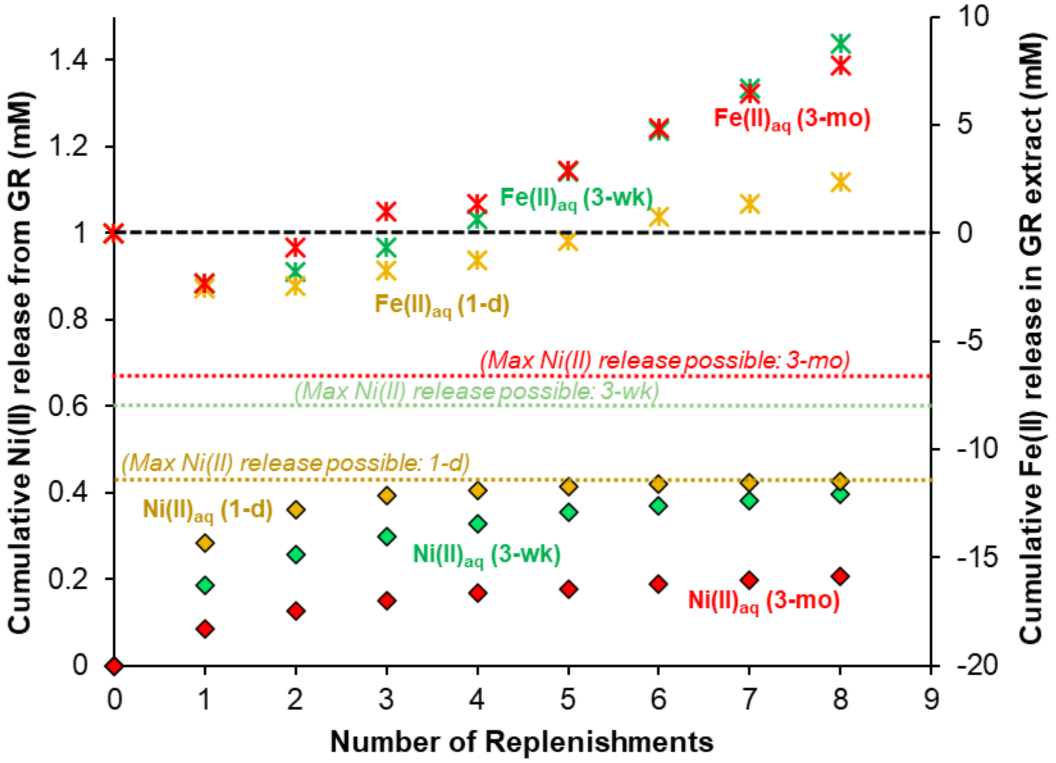

Figure S17. Cumulative Ni(II) and Fe(II) release from GR during sequential extractions with the pH 7.0 electrolyte. The left y-axis shows the cumulative concentration of $Ni(II)_{aq}$ released from GR, and the right y-axis shows the corresponding cumulative concentration of released $Fe(II)_{aq}$. The Ni(II)-GR sorption samples (aged for 1 day, 3 weeks, or 3 months) were subjected to eight consecutive replenishment steps. Colored diamonds represent $Ni(II)_{aq}$ release, and colored asterisks indicate $Fe(II)_{aq}$ concentrations. The black dashed line denotes the baseline corresponding to no net change in $Fe(II)_{aq}$ concentration. Colored dotted lines indicate the maximum cumulative concentration of released Ni(II) possible for each GR sample based on their total initial Ni content. The results show that Ni(II) release decreases markedly with GR aging, indicating Ni(II) retention within the deeper regions of GR particles. Standard deviations for duplicate samples at all data points were <2%; error bars are smaller than the symbol size and therefore not shown.

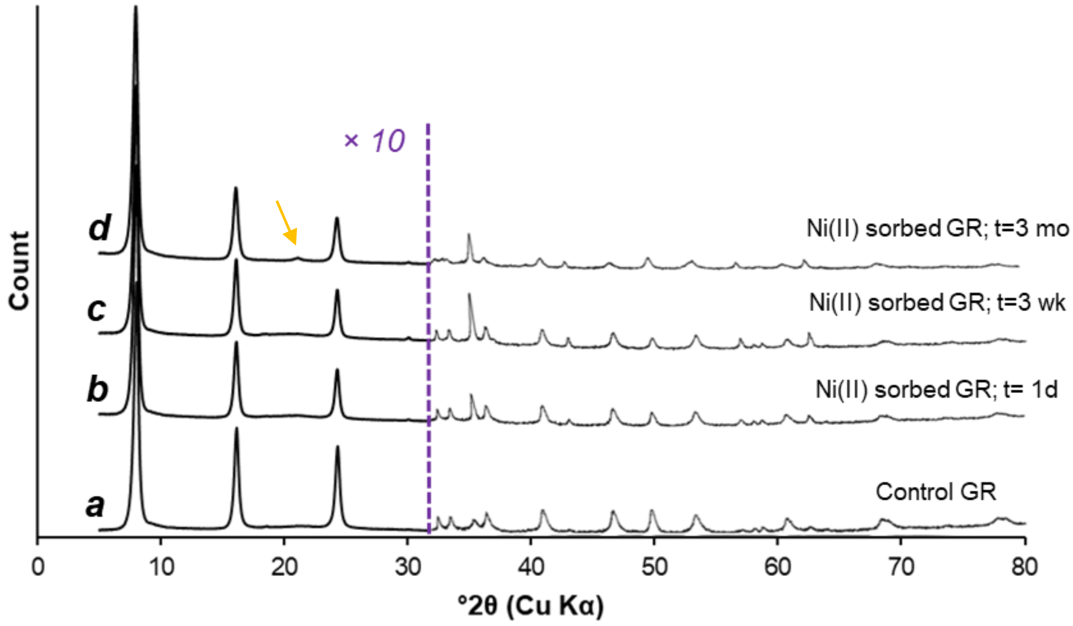

Figure S18. The background-subtracted and normalized XRD patterns of control GR (pattern a) and Ni(II)-sorbed GR (patterns b, c, and d) after 20 and 8 extraction cycles, respectively. The characteristic reflections of GR[2, 3] from the basal planes at low 2θ, and hkl bands in the high-angle sides are present all the GR samples, indicating that the bulk GR mineralogy has been preserved during the extraction series. The strongest peaks at 2θ = ~ 8°, 16°, and 24° correspond to the basal reflections resulting from the layered structure of GR. The regions to the high-angle side of the purple colored-dashed lines were scaled by a factor of 10 to enhance the visibility of the hkl bands at 32-80 °2θ. A small reflection at ~22° 2θ, marked by the yellow arrow, indicates minor precipitation of goethite in the 3-month extracted sample.

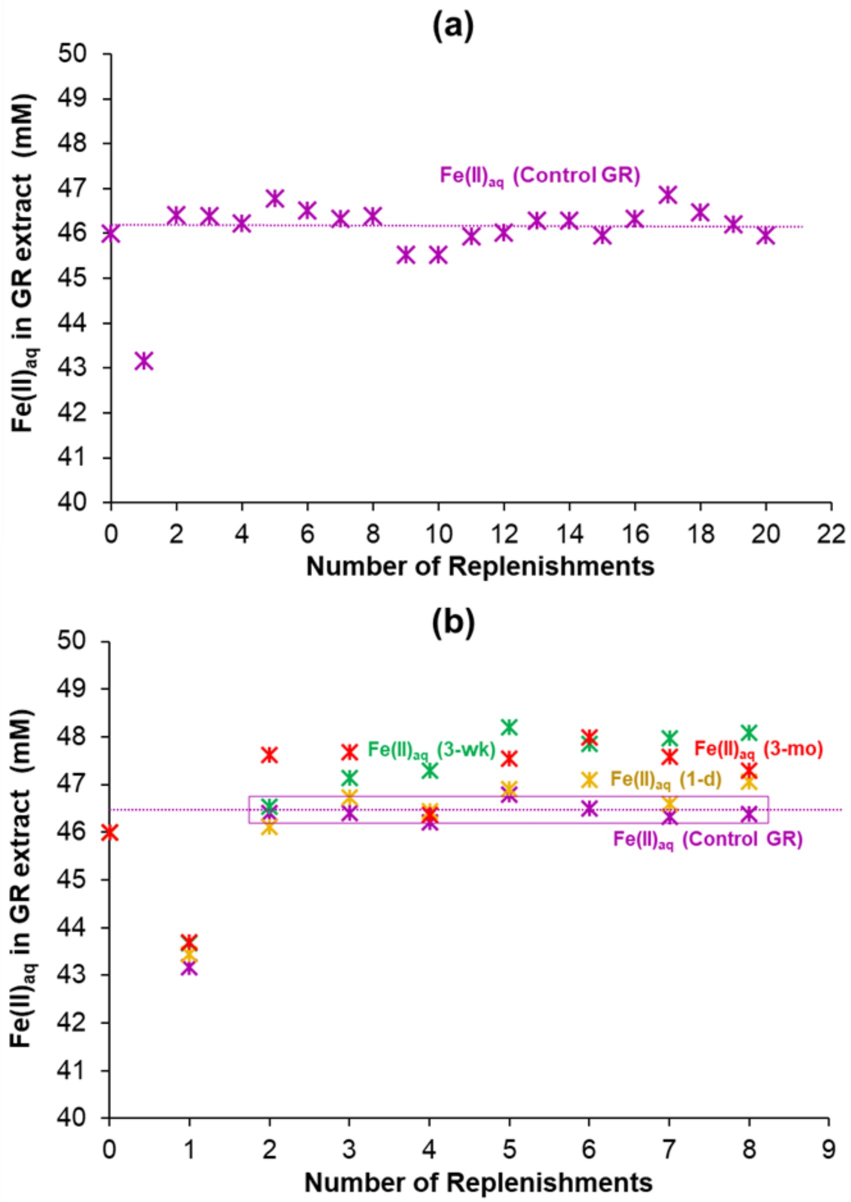

Figure S19. (a) $Fe(II)_{aq}$ concentrations in extractants of GR control during 20 consecutive replenishments with the extractant solute (pH 7.0 electrolyte solution). The purple dotted line shows the average $Fe(II)_{aq}$ concentration of the 20 replenishment steps, illustrating relatively stable $Fe(II)_{aq}$ levels with minor fluctuations around ~46 mM (< 4%); **(b)**, $Fe(II)_{aq}$ concentrations in extractant solutes from Ni(II)-GR sorption samples aged for 1 day, 3 weeks, and 3 months, and the control. The purple dotted line represents the average $Fe(II)_{aq}$ concentration measured for control GR from replenishment steps 2 through 8, and the purple box highlights the corresponding range of $Fe(II)_{aq}$ values. The $Fe(II)_{aq}$ concentrations in the Ni(II)-sorbed GR samples substantially exceed the values observed for control GR, indicating that Ni(II) incorporation induced GR dissolution by the extractant.

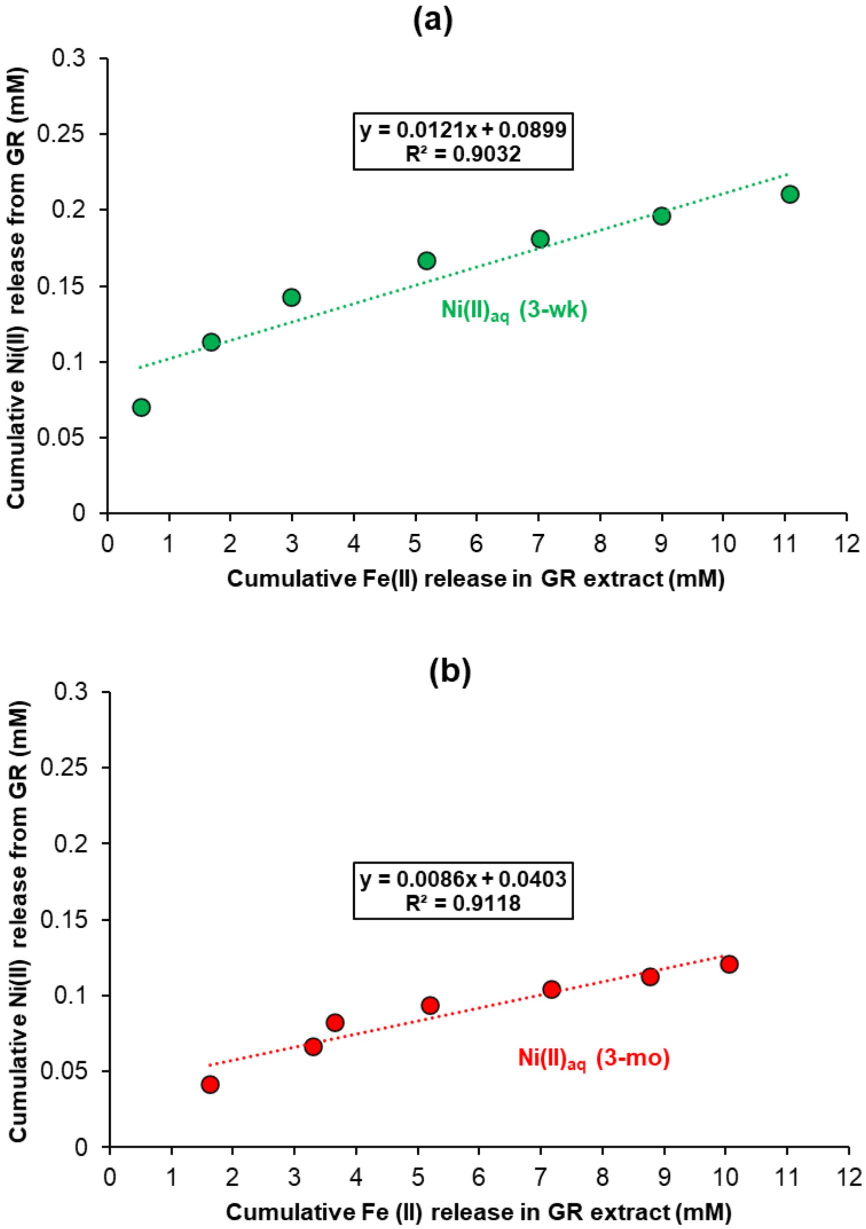

Figure S20. Linear correlations between cumulative Ni(II) and Fe(II) release from GR during sequential extractions from step 2 onward are presented for 3-week and 3-month aged Ni(II)-sorbed GR samples, respectively in panel (a) and (b). Both samples show strong linear relationships, with R^2 values of 0.9032 (3-wk) and 0.9118 (3-mo), indicating a linear relationship between Ni(II) and Fe(II) release consistent with a mostly homogeneous distribution of Ni(II) in the mineral matrix dissolved by the extractant.

REFERENCES

(1) Refait, P.; Géhin, A.; Abdelmoula, M.; Génin, J.-M. R. Coprecipitation Thermodynamics of Iron(II–III) Hydroxysulphate Green Rust from Fe(II) and Fe(III) Salts. *Corros. Sci.* **2003**, *45* (4), 659–676. https://doi.org/10.1016/S0010-938X(02)00138-5.

(2) Elzinga, E. J. Mechanistic Study of Ni(II) Sorption by Green Rust Sulfate. *Environ. Sci. Technol.* **2021**, *55* (15), 10411–10421. https://doi.org/10.1021/acs.est.1c01442.

(3) Alam, K. M. N.; Elzinga, E. J. Dynamics and Mechanisms of Mn(II), Co(II), Ni(II), Zn(II), and Cd(II) Sorption onto Green Rust Sulfate. *Environ. Sci. Technol.* **2023**, *57* (22), 8396–8405. https://doi.org/10.1021/acs.est.3c01584.

(4) Ressler, T. WinXAS: A New Software Package Not Only for the Analysis of Energy-Dispersive XAS Data. *J. Phys. IV* **1997**, *7* (C2), C2-269.

(5) Ravel, B.; Newville, M. ATHENA, ARTEMIS, HEPHAESTUS: Data Analysis for X-Ray Absorption Spectroscopy Using IFEFFIT. *J. Synchrotron Radiat.* **2005**, *12* (4), 537–541. https://doi.org/10.1107/S0909049505012719.

(6) Ankudinov, A. L.; Rehr, J. J. Relativistic Calculations of Spin-Dependent x-Ray-Absorption Spectra. *Phys. Rev. B* **1997**, *56* (4), R1712–R1716. https://doi.org/10.1103/PhysRevB.56.R1712.

(7) Ressler, T. WinXAS: A Program for X-Ray Absorption Spectroscopy Data Analysis under MS-Windows. *J. Synchrotron Radiat.* **1998**, *5* (2), 118–122. https://doi.org/10.1107/S0909049597019298.
